# An exploration of the political, social, economic and cultural factors affecting how different global regions initially reacted to the COVID-19 pandemic

**DOI:** 10.1098/rsfs.2021.0079

**Published:** 2022-02-11

**Authors:** Julian W. Tang, Miguela A. Caniza, Mike Dinn, Dominic E. Dwyer, Jean-Michel Heraud, Lance C. Jennings, Jen Kok, Kin On Kwok, Yuguo Li, Tze Ping Loh, Linsey C. Marr, Eva Megumi Nara, Nelun Perera, Reiko Saito, Carlos Santillan-Salas, Sheena Sullivan, Matt Warner, Aripuanã Watanabe, Sabeen Khurshid Zaidi

**Affiliations:** ^1^ Respiratory Sciences, University of Leicester, Leicester, UK; ^2^ St Jude Children's Research Hospital, Memphis, TN, USA; ^3^ British Antarctic Survey Medical Unit, Emergency Department, University Hospitals Plymouth NHS Trust, Plymouth, UK; ^4^ NSW Health Pathology - Institute for Clinical Pathology and Medical Research, and University of Sydney, Westmead, New South Wales, Australia; ^5^ Virology Department, Institut Pasteur de Dakar, Dakar, Senegal; ^6^ Department of Pathology and Biomedical Science, University of Otago, and Canterbury Health Laboratories, Christchurch, New Zealand; ^7^ JC School of Public Health and Primary Care, The Chinese University of Hong Kong, Hong Kong Special Administrative Region, People's Republic of China; ^8^ Stanley Ho Centre for Emerging Infectious Diseases, The Chinese University of Hong Kong, Hong Kong Special Administrative Region, People's Republic of China; ^9^ Hong Kong Institute of Asia-Pacific Studies, The Chinese University of Hong Kong, Hong Kong Special Administrative Region, People's Republic of China; ^10^ Shenzhen Research Institute of the Chinese University of Hong Kong, Shenzhen, People's Republic of China; ^11^ Department of Mechanical Engineering, The University of Hong Kong, Hong Kong Special Administrative Region, People's Republic of China; ^12^ Laboratory Medicine, National University Hospital, Singapore, Singapore; ^13^ Civil and Environmental Engineering, Virginia Tech, VA, USA; ^14^ Instituto de Investigaciones en Ciencias de la Salud, Universidad Nacional de Asunción, San Lorenzo, Paraguay; ^15^ Clinical Microbiology, University Hospitals of Leicester NHS Trust, Leicester, UK; ^16^ Division of International Health, Niigata University, Niigata, Japan; ^17^ Hospital Niño San Borja, Lima, Perú; ^18^ WHO Collaborating Centre for Reference and Research on Influenza, Royal Melbourne Hospital, Melbourne, Australia; ^19^ Department of Infectious Diseases, University of Melbourne at the Peter Doherty Institute for Infection and Immunity, Melbourne, Australia; ^20^ Department of Parasitology, Microbiology and Immunology, Federal University of Juiz de Fora, Juiz de Fora, Brazil; ^21^ Karachi Institute of Medical Sciences affiliated with National University of Medical Sciences, Karachi, Pakistan

**Keywords:** COVID-19, SARS-CoV-2, pandemic response, lockdown, government, guidance

## Abstract

Responses to the early (February–July 2020) COVID-19 pandemic varied widely, globally. Reasons for this are multiple but likely relate to the healthcare and financial resources then available, and the degree of trust in, and economic support provided by, national governments. Cultural factors also affected how different populations reacted to the various pandemic restrictions, like masking, social distancing and self-isolation or self-quarantine. The degree of compliance with these measures depended on how much individuals valued their needs and liberties over those of their society. Thus, several themes may be relevant when comparing pandemic responses across different regions. East and Southeast Asian populations tended to be more collectivist and self-sacrificing, responding quickly to early signs of the pandemic and readily complied with most restrictions to control its spread. Australasian, Eastern European, Scandinavian, some Middle Eastern, African and South American countries also responded promptly by imposing restrictions of varying severity, due to concerns for their wider society, including for some, the fragility of their healthcare systems. Western European and North American countries, with well-resourced healthcare systems, initially reacted more slowly, partly in an effort to maintain their economies but also to delay imposing pandemic restrictions that limited the personal freedoms of their citizens.

## 1. Introduction

On 30 January 2020, the World Health Organization (WHO) declared that COVID-19 caused by a novel coronavirus (SARS-CoV-2) constituted a Public Health Emergency of International Concern (PHEIC) [1], then on 11 March 2020, declared this to be a pandemic [2]. In response to these declarations, there were stark contrasts in how different countries and regions then managed the COVID-19 pandemic, with some countries taking it seriously and reacting immediately and comprehensively, and others adopting a wait and see attitude—with very different consequences.

Despite their high rankings on the Global Health Security Index, some of these countries (like the USA and UK—ranked 1 and 2 overall, respectively) performed surprisingly badly in terms of total COVID-19 case numbers and deaths when compared with others that scored much lower (like Vietnam and China—ranked 50 and 51 overall, respectively) [3].

### 1.1. How well did different regions manage to control the spread of SARS-CoV-2?

If we are to consider the feasibility of a more global consensus (or a more tiered and stratified) and collaborative approach to managing the next pandemic, the underlying reasons for these differences need to be understood more clearly. This review examines the early part of the COVID-19 pandemic (February–July 2020) and aims to explore various aspects of the early pandemic responses to help us understand how governments and their populations can work together better to limit the spread of the next pandemic pathogen.

During the first wave of the pandemic (February–July 2020), it is now well-recognized that some countries and jurisdictions in the East (Japan, Taiwan, South Korea, Hong Kong) and Southeast (Vietnam, Thailand, Singapore) Asia, and Australasia (Australia, New Zealand) reacted more quickly, comprehensively, and effectively than Western European countries and the Americas. Some Scandinavian (Norway, Finland, Denmark, Iceland) and Central and Eastern European countries (Hungary, Slovakia, the Czech Republic, Romania, Poland, Bulgaria, Ukraine) also managed to control the virus well, reporting fewer than 500 new cases per day—though early figures may be underestimates due to limited testing capacity. However, some neighbouring countries, like Belarus and Russia, fared worse, experiencing much higher daily cases numbers, similar to some of the Western European countries [4].

East and Southeast Asian countries also reacted earlier and comprehensively to news of the new mysterious pneumonia coming out of China, likely as a result of their experience with the 2003 SARS-CoV-1 outbreaks, the various, sporadic avian influenza—particularly A(H5N1) and A(H7N9)—outbreaks and the large MERS-CoV outbreak in South Korea in 2015.

Compared to the North American and some Western European developed nations, Australia and New Zealand have fared the best, overall, in terms of cumulative numbers of cases and deaths per million population, as of 1 July 2020 (rounded to nearest whole number) [4], e.g.

Australia: total cases 7920; total deaths 104; total population approximately 25 million; total cases/million 317; total deaths/million 4

New Zealand: total cases 1528; total deaths 22; total population approximately 5 million; total cases/million 306; total deaths/million 4

USA: total cases 2 849 111; total deaths 132 200; total population approximately 333 million; total cases/million 8556; total deaths/million 397

Canada: total cases 104 271; total deaths 8615; total population approximately 38 million; total cases/million 2744; total deaths/million 227

UK: total cases 283 372; total deaths 40 553; total population approximately 68 million; total cases/million 4167; total deaths/million 596

**Figure 1.**
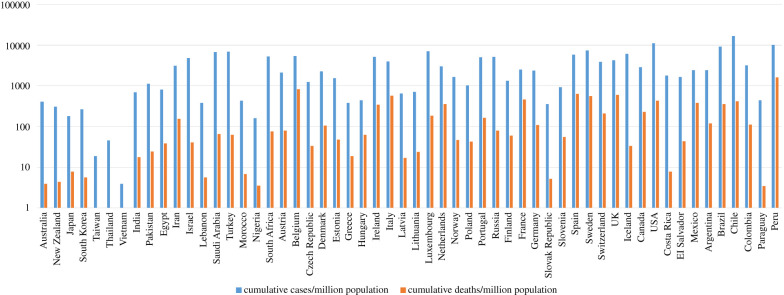
Cumulative COVID-19 cases and deaths for the early part of the pandemic (15 February–15 July 2020) for some example populations in the regions discussed in the main text (as extracted from Worldometer: https://www.worldometers.info/coronavirus/). NB: any values below 1.0 (cumulative deaths/million population: Taiwan, Thailand, Vietnam) have been normalized to 1.0 for plotting purposes.

Figure 1 shows the cumulative COVID-19 cases and death numbers during the early part of the pandemic (15 February–15 July 2020) for various countries that are covered in this article.

Although both Australia and New Zealand experienced peaks of COVID-19 cases in the first wave during March–May 2020, as did most other countries, New Zealand quickly shut down international travel and suppressed the virus continuously thereafter. COVID-19 disease was not uniformly distributed in Australia at the beginning of the pandemic, with Western Australia seeing relatively few cases compared to Victoria and New South Wales (NSW), which experienced a larger, longer second wave during its winter season (June–September 2020). More stringent measures suppressed the virus better until a third wave surge starting in June 2021, which was still ongoing as of 2 August 2021.

In East/Southeast Asia, Taiwan, Vietnam and Thailand reported less than 100 cases daily during the first year of the pandemic due to a rapid rollout of mass testing, universal masking and enforced isolation, border closure and quarantine [5–7]. The remaining countries listed above: Japan, Singapore, Hong Kong, South Korea, Australia and New Zealand, experienced some early spikes in daily cases numbers (but less than 2000), before bringing their epidemics under control. More recently (June 2021), some of these countries (e.g. Thailand and Japan) have been reporting higher daily cases numbers (5000–10 000), as new SARS-CoV-2 variants circulate through these largely non-immune and unvaccinated populations [4].

During March–June 2020, some of the larger South Asian countries (India, Sri Lanka, Nepal) did not report many laboratory-confirmed cases of COVID-19, whereas others (Pakistan, Bangladesh) reported several thousand daily cases [4]. Part of this is likely due to variations in testing capacity and sampling strategy during the first pandemic wave, with India reporting up to 100 000 new cases a day, and the other countries reporting several thousand new cases daily. Varying experience between East/Southeast Asian and South Asian countries may account for this difference, with the East/Southeast Asian countries also having a better healthcare infrastructure overall, together with their experience of SARS in 2003, and having to manage ongoing zoonotic threats from avian influenzas and other imported pathogens such as MERS-CoV (South Korea). South Asian countries do have their own sporadic, endemic virus threats, such as Nipah and Kyasanur Forest Disease viruses in India, where this experience, such as in Kerala with Nipah virus, has helped the local public health teams manage local COVID-19 outbreaks more effectively [8]. However, overall, the control of SARS-CoV-2 has been less effective in the South Asian versus the East/Southeast Asian countries during the early part of the pandemic.

Middle Eastern and North African (MENA) countries like Iran, Saudi Arabia, Turkey and Egypt showed similar patterns of reported COVID-19 cases, initially peaking sometime during March–June 2020. Iraq's COVID-19 pandemic seemed to start later in June 2020, and similarly, Israel and Lebanon reported very few daily cases during this first pandemic wave period. In some countries, this may be due to the rapid implementation of non-pharmaceutical interventions (NPIs) as well as limited testing capacity during this time [9], with any subsequent surges in case numbers likely due to religious or festive gatherings that the people and governments were reluctant to completely suppress [10,11]. There have also been long-standing issues around the transparency and availability of more detailed epidemiological data, such as age- and sex-stratified incidence and mortality figures, not only for COVID-19, but other infections, like HIV, which may have hampered the effectiveness of public health measures. Iraq and Lebanon currently offer the most, and Egypt and the United Arab Emirates (UAE) the least detailed epidemiological data in this context [12].

Despite most countries showing relatively sparse data, the timing of the pandemic in Africa is similar to that in Europe. This is most likely due to the frequent travel between these continents, as a consequence of a linked colonial past, modern day tourism and the fact that Europe is now Africa's largest trading partner [13]. Data from Algeria, Nigeria, South Africa, Kenya and Sudan all show that COVID-19 cases were present in April 2020, with a first wave extending to August–September 2020 [4].

Western European countries were generally slow to take the pandemic seriously, with early efforts mostly focused on giving overseas aid rather than reviewing and consolidating their own pandemic preparedness, such as the sending of shipments of personal protective equipment (PPE) to China during January-March 2020, while the pandemic was spreading across Europe [14,15]. In addition, a lot of time was wasted as individual countries debated around the evidence of how the virus was predominantly transmitted, the effectiveness of masks, an over-emphasis on handwashing, and ultimately, whether the virus was airborne or not and what the appropriate level of PPE should be in light of this uncertainty for healthcare workers, and later on, the general public.

Thus, Western Europe and North America experienced devastating first waves of COVID-19 during March–June 2020. Much of the spread within Western Europe was initially driven by the winter skiing season, as the virus was exported from East/Southeast Asia to European ski resorts, from where it was then imported back into the UK and other European skiers' hometowns [16].

As the most popular ski resorts in Europe lay in Western Europe, the Central/Eastern European countries (e.g. Hungary, Poland, Romania, the Czech Republic, Bulgaria, Slovakia, Slovenia and Ukraine) may have been spared much of this early spread that led to the first wave. As a result, in stark contrast to the Western European countries, the COVID-19 case numbers in Central/Eastern European countries remained well under 1000 cases daily, with fewer than 100 deaths per day, during the first wave of the pandemic in March–July 2020 [2].

Like the UK and Europe, the US and Canada experienced their first wave of cases in March–June 2020. In the first few months, the availability of testing was limited, and the US Centers for Disease Control (CDC)'s own nucleic acid amplification test (NAAT) was initially flawed. The US response was particularly hampered by a leadership that was sceptical of the reality of the pandemic and sidelined the CDC and health experts [17]. Public health messaging was confusing and contradictory: ‘the virus is just a flu and not serious' [18], and later on, ‘it's not airborne, just wash your hands' [19], ‘masks don't work, don't buy them’, etc. [20].

Even after the effectiveness of masks was established, the US President mocked them and turned them into a political symbol [21]. The US should have been one of the countries that was best prepared to handle a pandemic because of its well-regarded public health agency and its scientific and healthcare resources, yet it had one of the highest mortality rates compared to other large, high-income OECD countries [22]. This may be due in part to the individually state-governed healthcare systems in the US that may have led to fragmented COVID-19 data reporting to the US CDC, with subsequent delays in implementing appropriate public health interventions in the hardest hit areas.

In Mexico, cases grew more slowly, starting in March 2020. There, the leadership also downplayed the impact of the pandemic and questioned medical expertise [23]. Elsewhere in Central and South America, the first wave of the epidemic—at least that recorded by the available testing at that time—appeared to start later, lasting from April–May to August–October 2020, with most of the larger countries having daily case counts never dropping below several thousand cases a day, and often being much higher [4]. This may also be linked to available testing capacity at that time, with countries like Brazil, Peru and Mexico reporting rising daily COVID-19 case numbers during April, and other countries like Argentina, Colombia, Venezuela and Guatemala only starting to report rising case numbers in May 2020 [4].

With notable exceptions in Central/Eastern Europe and Scandinavia, overall, in many Western hemisphere countries, there was a slow realization about the seriousness of the pandemic. This was coupled with limited capacity and experience in rolling out mass testing programmes, and the linking of positive results to the isolation of positive cases, and the quarantining of their contacts. In addition, they were particularly slow to recognize and understand that the virus was spreading in a more rapid manner that was more consistent with aerosols than droplet, contact or fomite routes [24]. The long debates and hesitation in imposing national lockdowns, the effectiveness of which were initially demonstrated by China and some other East/Southeast Asian countries, also allowed the virus to spread further and faster in these Western nations. This resulted in most Western nations having experienced, globally, the worst first wave of the pandemic, in terms of COVID-19 case numbers and deaths—particularly in the elderly, during March–June 2020.

## 2. Aims and objectives

Although many articles have examined what interventions were put in place and their relative effectiveness [25–27], there has been less written about why some interventions were used and not others by different countries, but more importantly, why some were implemented earlier in some countries than others—and also the underlying factors influencing the level of population compliance with them [28]. This article aims to identify the factors impacting on how and why the early part of the pandemic was handled differently by different countries—mostly using NPIs—and the way that governments and their populations collaborated (or not) in this, to control the spread of the virus. It draws freely on both academic and media publications, as news and trends on the rapid spread of the pandemic relied heavily on real-time media articles, many of which were rigorously fact-checked for accuracy—particularly around contemporaneous social and political attitudes to pandemic-related topics.

The idea behind this is that for any new pandemic pathogen, antimicrobial agents and vaccines will take several months to develop and mass-produce, and it is during this early phase of any pandemic that NPIs will play a crucial role in limiting the spread of the pathogen. This gives sufficient time to build capacity for increasing laboratory testing, hospital ward and intensive care beds, and PPE supplies. This in turn allows time for the development and trialling of new antiviral drugs and vaccines, then the manufacturing, delivery and administration of successful candidates at scale. This would also need reciprocal border control policies between countries and determining equitable rules to govern this across multiple international jurisdictions, the mechanisms and practicalities of which are beyond the scope of this article.

The rest of the article will examine in more detail how populations from various countries from each of these regions reacted during the first wave of the COVID-19 pandemic and complied with their governments' various NPI recommendations to reduce the transmission of SARS-CoV-2. These included the variable use of masks and social distancing (including limited or full local and national lockdowns and curfews), together with a qualitative impression of various countries’ capacities to perform SARS-CoV-2 laboratory testing, isolate confirmed COVID-19 cases, and quarantine their contacts, as indicated by the headings below:

**Government trust:** how much did the population believe their government's COVID-19 advice?
**Testing capacity**: how much SARS-CoV-2 testing did they do in the hospitals and the community?
**Track/Isolation/Quarantine:** how effectively did they successfully trace and isolate COVID-19 cases, and quarantine their contacts?
**Compliance with social distancing restrictions:** how closely did the population comply with their government COVID-19 guidance?
**Masking:** how well did the population accept and follow their government mask mandates?
**Overall effectiveness of SARS-CoV-2 control:** how well did the country control the spread of SARS-CoV-2?

Understanding different countries' cultural, economic, social factors involved in their early COVID-19 pandemic response can help us identify modifiable factors that can lead to a more effective regional and global response protocol for the next pandemic.

Note that this exploratory review is an attempt to describe and understand the populations’ responses to their various governments' policies during the early pandemic. To this end, some mention of those government policies is made, but the main emphasis is more on assessing the underlying factors in these populations that led to the degree to which these populations adhered to these policies—rather than to compare how and why these governments arrived at these policies per se.

### 2.1. Australasia

**Government trust:** generally high

**Testing capacity:** initially limited, but rapidly increased

**Track/Isolation/Quarantine:** rapid expansion of capacity, strongly enforced

**Compliance with social distancing restrictions:** high to very high

**Masking:** not initially universally mandated

**Effectiveness of SARS-CoV-2 control:** very good to excellent

Australia and New Zealand have fared the best out of all the North American and Western European developed nations in terms of COVID-19 case numbers and deaths. Both score at the top level of 5 (out of 5) on the WHO COVID-19 Preparedness and Response Plan (CPRP, where 1 = no capacity and 5 = sustainable capacity) [29]. Australia and New Zealand also have a geographic advantage in terms of their isolation, where they share no land borders with any other nations.

How did they manage this? Several factors have likely contributed to this, including a shared and heightened awareness of emerging zoonotic virus threats with neighbouring countries in East/Southeast Asia. Both countries had pre-existing pandemic plans.

*Australia's Health Management Plan for Pandemic Influenza* (AHMPPI) dated back to 1999, which was later updated in 2014 in response to the 2009 A(H1N1)pdm09 influenza pandemic, with a further minor update in August 2019. *New Zealand's Influenza Pandemic Plan* (NZIPP) dated from slightly earlier in 1999 [30–33].

New Zealand was the first country to test its plan with a National Exercise ‘Virex’ in 2001, after which the *New Zealand Influenza Pandemic Action Plan* (NZIPAP) was developed in 2002. This has undergone substantial revision since then due to the evolving threat from avian A(H5N1) influenza, the influenza A (H1N1) 2009 influenza pandemic, and the subsequent all-of-government programme of pandemic planning and exercises implemented in August 2017 [30–33]. Other developed Western countries, like the UK, also produced pandemic plans, such as a previous coronavirus pandemic plan written in 2005 after the 2003 SARS outbreaks. Yet this went ‘missing’ somewhere in Whitehall [34]. Clearly, a regular review process of such pandemic plans, during inter-pandemic periods, is required to keep them updated and accessible as and when needed.

Both Australia and New Zealand closed their borders to foreigners (and even to their citizens in Australia, [35]) in March 2020, with a 14-day quarantine mandated for any new arrivals. A rapid expansion of testing facilities to support rapid and effective test and trace systems led to the rapid identification and screening of any infected individuals and their contacts. Any resulting potential for local community spread was met with a rapid implementation of tiered local and larger regional or national lockdowns [36,37]. However, initially, neither country mandated the wearing of masks, though this changed later to become more widespread in Australia as the pandemic evolved [38,39].

In addition, very early on, New Zealand made the decision to go for a ‘virus elimination’ policy, i.e. to suppress virus transmission completely. This involved aggressive, early intervention with lockdown ‘stay-at-home’ orders, school and university closures, shutting of non-essential businesses, supply rationing and extensive restrictions on travel, substantial financial support packages, along with contact tracing, extensive testing and use of a smartphone COVID tracer app. This approach worked, with New Zealand claiming that the country was COVID-19-free by early June 2020 [30]. Although New Zealand had not experienced an outbreak of a novel pathogen previously, its emergency services, decisive governance, effective communication and high population compliance were already primed through past experience of public health emergencies, such as earthquakes [40].

Australia did not take the same ‘virus elimination’ approach, but rather more of a ‘flattening the curve’ strategy, where more suppressive measures would be applied only when case numbers were rising. These included stay-at-home orders, bans on social gatherings of more than two people, severe local travel restrictions, testing and tracing (including a smartphone app—‘COVIDsafe’—though this was not particularly successful), and 14-day quarantines for foreign travellers arriving in the country.

Additional support measures included economic support such as a ‘JobKeeper’ allowance to retain workers, childcare finance relief, increased payments to those on welfare, and financial stimuli to help banks lend to struggling businesses. Efforts were also made to communicate more effectively to diverse ethnic groups to improve their understanding and compliance with contemporary pandemic-related restrictions—but also with any future healthcare needs in these culturally diverse communities [41].

Although Australia, unlike New Zealand, experienced a second wave in one state (Victoria) and an ongoing low level of COVID-19 cases across the country, it was still very successful in controlling COVID-19 cases and deaths compared to other Western European and North American countries [30].

One notable setback in Australia's early pandemic control efforts included the importation of COVID-19 cases from the cruise ship ‘Ruby Princess’, which docked in Sydney on 19 March 2020, following an 11-day cruise between Sydney and New Zealand. All 2700 passengers were allowed to disembark, with 100 of them feeling unwell. This resulted in 900 additional COVID-19 cases, 28 of whom died [42].

The key behind the success of these two countries was the high level of support for and compliance with their governments' actions—particularly border closures—with both Australian and New Zealand government scoring 70–80% approval ratings for their COVID-19 strategies [35–37]. This, again, may be related to their long-standing heightened awareness of possible novel zoonotic viral threats to both humans and their livestock and agriculture, but also due to their close proximity to East/Southeast Asia—an influence that the other English speaking countries (Canada, USA and UK) lacked. Whilst New Zealand has a single, centralized, top-down chain of government command, in Australia, it is the individual state premiers who manage the daily practicalities of the pandemic. It is notable that both styles of government in these countries managed to effectively control the spread of the virus during the early pandemic.

### 2.2. Asia

**Government trust:** very variable—low to high

**Testing capacity**: initially limited, but rapidly increased, particularly in East/Southeast Asia

**Track/Isolation/Quarantine**: rapid expansion of capacity, strongly supported and enforced in East/Southeast Asia; less so in South Asia

**Compliance with social distancing restrictions:** high to very high in East/Southeast Asia; low to moderate in South Asia

**Masking:** mostly universal and voluntary in East/Southeast Asia; usually less than 50% in South Asia

**Effectiveness of SARS-CoV-2 control:** very good to excellent in East/Southeast Asia; low to moderate in South Asia

Asia is credited with both the origin of SARS-CoV-2, as well as examples of some of the most effective COVID-19 pandemic national control programmes, globally. The appearance of clusters of pneumonia in Wuhan (capital of Hubei province, China) in late December 2019 and early January 2020 did not trigger an immediate national lockdown. Some Chinese New Year-related travel was ongoing until the first lockdown in Wuhan on 23 January 2020, followed by travel restrictions in other nearby cities. By this time COVID-19 cases in Thailand, Japan and South Korea had already been reported [43–46].

An initial meeting by the WHO on 22 January 2020 did not initially consider this novel coronavirus as posing an international threat. It was not until 30 January, just after early cases were reported from Europe [47], that the WHO declared this novel coronavirus a PHEIC [1].

Once the other East and Southeast Asian countries became aware of the emerging novel coronavirus threat, their responses were mostly swift, decisive and unified [48], with tight border controls and mandatory 14-day quarantines for all foreigners entering the country; universal masking with governments supplying the population with masks; a rapid expansion of testing with accompanying tracing of new positive cases; enforced isolation of infected cases and quarantine of their contacts—using phone tracking apps and security tags, in some cases.

Among more than 70 000 confirmed COVID-19 cases in China by 11 February 2020, nearly 75% were found in the province of Hubei, with its capital city of Wuhan [49]. This massive number of cases required drastic action to isolate the infected and quarantine their contacts, to control the spread of the virus. To this end, a new 1000-bed hospital (the Huoshenshan hospital in Wuhan) was built in 10 days [50]. To staff the new hospital, manpower and other resources were mobilized from elsewhere in the country, including nearly 20 000 nurses by 1 March 2020 [51]. Masks, goggles and protective clothing were also sent by Japanese cities to their sister cities in China [52].

In Hong Kong, high levels of perceived risk related to COVID-19 amongst the general population was observed during the early phase of pandemic [53]. This was reflected in the Hong Kong Government's adoption of a containment strategy consisting of early identification, isolation and treatment of COVID-19 cases [54,55]. A high level of public cooperation in the voluntary adoption of individual protective measures, such as enhanced personal hygiene, masking, the avoidance of unnecessary travel, and rigorous social distancing, all helped to control the spread of SARS-CoV-2 during the early pandemic [56–58].

These examples, in the cities of Wuhan and Hong Kong, demonstrated how effective virus control can be achieved by early and stringent public health interventions [28]. It is of note that the health authorities in China already suspected possible aerosol transmission of SARS-CoV-2 as early as 18 February 2020 [59].

Many of these East/Southeast Asian jurisdictions became ‘exemplars’ of the early COVID-19 pandemic responses, keeping case numbers and deaths to a minimum, including Taiwan [60], Hong Kong [56], Singapore [61], Malaysia [62], Philippines [63], Vietnam [64], Thailand [65], Japan [66] and South Korea [67]. All these countries score 3–5 on the WHO's CSPRP scale, e.g. with Malaysia scoring 5 and Indonesia and the Philippines scoring 3, indicating that most of their healthcare systems in the region could maintain some degree of sustained effort in combatting the novel virus [29].

Several other factors differed from Western countries amongst these Asian populations that may also have contributed to their much lower COVID-19 case numbers and deaths. These include a more compliant population with enhanced awareness of their individual public health and social responsibilities (e.g. universal masking to both protect themselves and others), likely due to their experience with SARS-CoV-1, avian influenzas (A/H5N1, A/H7N9), MERS-CoV (South Korea) and a lower overall prevalence of obesity [68], a risk factor for more severe COVID-19.

However, there were some early ‘hiccups’ to the control of SARS-CoV-2 in some of these countries due to specific populations, such as the passengers and crew in the Diamond Princess Cruise ship in Japan [69], migrant workers in Singapore and members of a specific church in South Korea.

In 2019, there were about 1.4 million migrant workers in Singapore (about 25% of the total population). Such work includes construction (involving about 300 000 or 21% of such migrant workers), chemical, pharmaceutical and petroleum processing, and shipyard labour. Most of these migrant workers came from Bangladesh, India and Myanmar. COVID-19 cases in Singaporean migrant workers started to rise in late February and March 2020. During April to September 2020, they constituted over 90% of all Singapore's laboratory-confirmed COVID-19 cases. High density, crowded, cramped housing conditions in designated migrant worker dormitories are a likely cause for this spread [70–72].

Thus, migrant workers have been the cause of much SARS-CoV-2 transmission in Asia. The ejection of migrant workers and closing of borders by Thailand in the early pandemic likely drove the virus to neighbouring regions, as these migrant workers returned home to countries with relatively fragile healthcare systems (predominantly to Laos, Cambodia and Myanmar). Singapore's approach of confining migrant workers in specific locations led to explosive outbreaks in worker dormitories. In contrast, Vietnam managed migrant workers alongside their citizens with a system of tiered institutional (enforced) and home isolation and quarantine, as well as phone app surveillance systems and targeted (rather than mass) testing. In addition, businesses set up ‘rice ATMs’ to support those most in need who became unemployed when the pandemic struck. These ATMs provided up to 2 kg of free rice per visit, daily [73–75].

South Korea managed to control the number of COVID-19 cases very effectively since its first imported case, a 35-year-old Chinese woman who returned from Wuhan on 20 January, 2021. However, this changed with ‘Case 31’, who had visited multiple locations in Daegu and Seoul before and after the onset of her symptoms, before her official diagnosis on 17 February 2020. These locations included a hospital, hotel restaurant and services at the Shincheonji Church of Jesus in Daegu. Within days of the authorities confirming her as the 31st COVID-19 case in the country, hundreds of new cases linked to the church and surrounding areas were being confirmed. The Korean Centers for Disease Control and Prevention estimated that she had had over 1000 contacts and by 18 March 2020, it was estimated that over 60% of COVID-19 cases (approx. 5000 cases out of approx. 8000 in total) were linked to this church cluster [76,77].

Although some of these East/Southeast Asian jurisdictions are single-party states, several are also governed democratically. These include South Korea, Taiwan and Japan. So their success in the control of COVID-19 is not just a result of the type of government, but also the way these governments and their populations responded. In general, these East/Southeast Asian countries saw what was happening in China and reacted quickly and decisively with high compliance, to enact relatively severe public health measures to stop the virus spreading [48]. These countries demonstrated that once the spread of the virus is well-controlled by early, rapid and stringently implemented interventions, a few additional new positive cases each day can be managed very effectively—allowing their economies to reopen with little or no interruption. Australia and New Zealand also followed this approach with notable success.

Finally, the development of new diagnostic tests for this new coronavirus, globally, could not have been achieved so quickly without the early release of the virus whole genome sequence on 10 January 2020 by a Chinese team of researchers, in collaboration with their Australian colleagues [78].

Other countries in South Asia (India, Pakistan, Bangladesh, Sri Lanka, Nepal), which score 2–3 on the WHO's CSPRP scale [29], generally fared less well, with much higher daily COVID-19 case numbers and deaths compared to their East/Southeast Asian counterparts.

India reported its first COVID-19 case on 30 January (imported from China), with other early cases in the cities of Thrissur, Alappuzha and Kasargod, all in the state of Kerala. This prompted the Indian government to instigate a lockdown in Kerala initially on 23 March, followed by a nationwide lockdown on 25 March 2020, in an attempt to control virus spread. This nationwide lockdown was further extended twice to the end of May 2020. As well as widespread poverty, ongoing endemic diseases such as HIV and tuberculosis, and a fragmented under-resourced public and expensive private healthcare system, India also faced serious problems with the virus spreading amongst millions of migrant workers—many of whom were caught stranded when national lockdowns were imposed. Many were forced to walk hundreds of miles to return to their home villages, without access to adequate food and water en route. The Indian Railways laid on special ‘Shramik trains’ to help ferry migrant workers home, with about 1600 trains transporting more than 2 million workers home by the third week of May 2020. Despite this, the news at this time was still full of migrant worker deaths caused by road and train accidents, which made national and international headlines [79–81].

Like other South Asian countries, limited laboratory testing capacity—mostly targeted at symptomatic cases of suspected COVID-19—was a serious problem during the early pandemic, with initially only the National Institute of Virology (Pune, Maharashtra) equipped and authorized to perform molecular diagnostic (NAAT) testing for SARS-CoV-2 in January 2020. Close to the time of the first national lockdown in March 2020, only 6500 tests had been conducted, nationwide, and the daily testing capability by mid-March 2020 was only 1400 tests per day. Later, this testing capacity massively increased with over 1000 government and private laboratories enabling 200 000–300 000 tests daily by the end of June 2020. On top of all of this, India also had to cope with misinformation and alternative and ‘fake’ remedies being constantly offered for treating the virus—a pattern that would been seen globally, to lesser or greater extents, in other countries, disseminated by internet sources and social media [79–92].

By mid-May, about half of all reported COVID-19 cases were concentrated in the five largest cities of Mumbai, Delhi, Ahmedabad, Chennai and Thane [83,84]. However, these figures are likely underestimates, with India performing only around 200 000–300 000 tests per day by June 2020 for a population of almost 1.4 billion [84]. India also likely opened up too early in June 2020 [85], allowing a resurgence of the virus in August–September 2020 that reached a peak of 97 000 daily new cases, which only waned towards 20 000 daily new cases by the end of the year [86].

Pakistan, the next most populous country in the region showed a similar pattern, with their first COVID-19 case reported on 26 February (imported from Iran), then entering a nationwide lockdown on 1 April until 9 May 2020. Again, populations in their largest conurbations: Karachi, Lahore, Islamabad and Peshawar contained over half of all reported COVID-19 cases [87]. Unlike the populations in East/Southeast Asia, masking was low in both India and Pakistan, with 50% or lower compliance [88,89].

In Bangladesh, the first COVID-19 cases were identified on 8 March, which led to national lockdown measures during 23 March to 30 May 2020 [90]. As well as the usual social distancing requirements, masking was more prominent there, with ongoing campaigns by the Bangladesh Rural Advancement Committee (BRAC—the world's largest non-government organization) to reinforce this message [91], using a ‘NORMalize’ approach of **N**o-cost (free masks distributed door-to-door); **O**ffering information on mask-wearing by videos and leaflets; **R**einforcement of mask-wearing behaviour; **M**odelling the benefits of mask-wearing and endorsement from community leaders [92].

Applying these four actions, it was found that in a large randomized control study of 340 000 people in 600 villages in Bangladesh, mask-wearing increased to 42% in participating villages, a 13% increase compared to control villages. The research team also compared symptomatic COVID-19 incidence in participating and control villages, and found 9.3% fewer symptomatic infections than in the control villages, if wearing cloth masks. This reduction was greater at 11% overall if surgical masks were worn instead of cloth masks, and was even greater at 23% lower in the 50–60 year olds and 35% in those over 60 [93].

This study has been one of the largest trials to show this benefit, which reinforces the findings from other more epidemiological studies on impact of universal masking in Asia [56,57].

Sri Lanka reported its first COVID-19 case on 27 January 2020 (imported from Hubei, China) and initially imposed a variety of restrictions across the country from March to 11 May, 2020. During this period, the government of Sri Lanka initially introduced various sequential control measures such as island-wide school closure, travel bans on selected countries (South Korea, Italy and Iran), declaration of special holidays to limit public gathering, shutting down the Colombo International Airport for all arrivals to the island, before finally imposing an island-wide lockdown ‘curfew’ from June 2020 [94,95].

Although significant numbers of COVID-19 cases were not reached until September 2020 (only about 3000 cases in total had been reported by the end of August 2020) [96], it is likely that the testing capacity was a limiting factor, with total tests per million population lying somewhere between that of India and Pakistan, but higher than Bangladesh [96].

Sri Lanka also benefited from a strong government-supported public health response which entailed teams of public health personnel contact tracing and monitoring the self-isolation/quarantine of individual households [94,95]. Universal masking was not emphasized as much as other social distancing measures in the early pandemic, due to limited PPE supplies, though in later waves, Sri Lanka also developed its own mask manufacturing capacity [97,98]. Stronger, police-enforced mask mandates were also imposed later on as COVID-19 cases surged towards the end of April and early May 2020 [99,100]. The key to success in managing the first pandemic wave in Sri Lanka was really due to the strict law enforcement by the Sri Lankan Police and the Tri Forces (the Sri Lankan armed forces: army, navy and air force) on the isolation of every positive COVID-19 case and the quarantine of their contacts in special quarantine facilities run by the Sri Lankan Government. This was done at no cost to the individuals, but with heavy penalties if they defaulted.

The first COVID-19 case in Nepal (also the first case in South Asia—imported from Wuhan, China) was reported on 23 January 2020. This appeared to be an isolated event, giving time for the Nepalese government to expand their diagnostic testing and hospital capacity, and increase border screening and controls—particularly along the border with India. After a second imported case in a returning traveller from France on 23 March 2020, a much longer national lockdown starting 24 March was imposed, which eventually lasted until 21 July 2020 [101,102].

As with other low income countries, Nepal faced multiple problems during the early pandemic, with a somewhat ‘leaky’ border with India allowing migrant workers to import COVID-19 cases, an under-resourced healthcare system, and insufficient economic support for those forced to self-isolate/quarantine in crowded housing, or poorly resourced quarantine centres [103].

As with Sri Lanka, the early lack of PPE made both populations more dependent on social distancing measures, but eventually as supplies increased, both the Sri Lankans and Nepalese voluntarily increased their use of masks [104,105]. Both countries benefited from a generally younger population with far fewer elderly care homes than in western countries, which likely limited the COVID-19-related mortality rates seen in the early pandemic.

Overall, the responses of these five most populous nations in South Asia were less comprehensive and organized compared to their East/Southeast Asian counterparts. This can be partly explained by a lower level of health resources per capita (as indicated by the WHO's CSPRP scores) in these South Asian countries, as well as their lack of more direct experience with SARS 2003, avian influenzas (A/H5N1, A/H7N9) and MERS-CoV outbreaks, which have stimulated East/Southeast Asian countries to develop and maintain a well-resourced and highly responsive pandemic infrastructure.

### 2.3. Middle East and North Africa

**Government trust:** low to moderate

**Testing capacity**: initially poor, with limited capacity to expand in the North African countries

**Track/Isolation/Quarantine**: self-imposed and enforced due to limited hospital capacity

**Compliance with social distancing restrictions:** variable to high

**Masking:** variable across different countries

**Effectiveness of SARS-CoV-2 control:** variable to good—but limited testing capacity in North African countries makes this difficult to assess

Middle East and North African (MENA) countries generally moved rapidly to stringent NPI control measures following the highly publicized and dramatic outbreak of COVID-19 in Iran during March 2020 [106], and likely in response to the news of the early detection of the first case of COVID-19 in Egypt on 14 February 2020 [107]. Nearby countries, Iraq, Lebanon, UAE and Jordan, quickly imposed strict measures to limit social contact, including social distancing regulations, and the closing of schools and international borders. Several neighbouring countries soon introduced complete national lockdowns with curfews, and other MENA countries followed with most restrictions remaining in place during March and April 2020, or longer [108].

The North African countries Morocco and Tunisia also followed this trend, with early border and school closures, bans on public gatherings, strict lockdowns and curfews. In addition, these countries, along with Jordan, already had institutions in place designed to react to outbreaks of emerging infections: Tunisia's National Observatory of New and Emerging Diseases, and a newly formed National Coronavirus Response Authority, which coordinated responses between it and other committees; Jordan's National Committee for Epidemics, and another related group, the Coronavirus Crisis Cell that coordinated pandemic responses; Morocco and Lebanon, similarly created additional advisory committees. Yet all of these bodies faced communication and compliance issues with their local populations [109–112].

Some have called for a more uniform response to the pandemic [7], but this may be difficult due to the differences in resources and infrastructure in each country. Similarly, others have called for more disaggregated data to be made available across the region. This will improve the planning of public health interventions, and related messaging, as well as generally improving surveillance that will help to target resources where there is most need. This lack of publicly available, granular data predates the COVID-19 pandemic and potentially limits the effectiveness of public health measures across many areas [12].

As a result of these early, comprehensive responses, these MENA countries managed to control the virus relatively well, comparable to other successful countries, such as Australia and South Korea, during March–June 2020. Part of this success was due to the use of full lockdowns at various time points in specific areas, during the early first pandemic wave, such as in Jordon, Lebanon, Tunisia, Algeria, Saudi Arabia, Kuwait, Iran, UAE and Yemen, with milder restrictions like partial lockdowns and night curfews in Bahrain, Egypt, Libya, Iraq, Morocco, Oman, the Palestinian Authority, Qatar and Syria [113].

Masking was mandated in almost all of these countries in public areas and on public transport, except in Yemen and Syria. In addition, there was a requirement in nearly all these countries for incoming travellers to either self-quarantine, use a health-tracking app, or to be screened for COVID-19 on arrival. Only Algeria, the Palestinian Authority, Syria and Yemen had no such requirements. Some of these discrepancies may have been due to the need to allow the rapid and unimpeded movement of refugees, including the seeking of asylum, across specific borders where there were ongoing civil conflicts [113].

However, despite this early success, the impatience to reopen businesses and international borders to revive the economy in the summer of 2020 to promote trade and travel, resulted in second wave peaks of COVID-19 cases [109].

One of the barriers to a consistent and uniform response to the pandemic across the MENA region is that the quality of the healthcare systems vary widely, from those in the Gulf Cooperation Council (GCC) countries (Bahrain, Kuwait, Oman, Qatar, Saudi Arabia and the UAE) to those in the North African countries. For comparison, the GCC countries (except for Qatar) score 4–5 on the WHO CPRP scale [29], similar to those of the health systems in North American, the UK, Europe, Israel, Australia and New Zealand. On this same scale, however, most of the other developing countries in MENA score lower, with scores of 4 for Algeria, Egypt and Iran; 3 for Jordan, Qatar, Lebanon Algeria, Tunisia and Morocco; 2 for Iraq, Yemen, Syrian Arab Republic, Libya, Djibouti and the occupied Palestinian territories.

Part of the reason for this variability is that most of the developing MENA countries spend less on healthcare than other countries of similar income, which has resulted in many hospitals being understaffed and under-resourced—well below the WHO recommended levels of 4.45 doctors, nurses and midwives per 1000 population. In Morocco and Egypt, these numbers are 0.72 and 0.79, respectively. In addition, some ‘fragile’ countries such as Syria and Yemen have ongoing civil conflicts and movement of refugees, whereas Lebanon has had to deal with a deteriorating economy and a massive accidental explosion in the Port of Beirut (due to unstable stores of almost 3000 tonnes of ammonium nitrate fertilizer) that disabled most of the medical services in the city [114]. Iraq has also faced decades of childhood poverty and influxes of refugees from neighbouring Syria, with a chronically under-funded and under-resourced healthcare system, making the additional burden of the pandemic and the rapidly rising COVID-19 case numbers in August 2020 ‘alarming’ and constituting a ‘major health crisis’, according to the WHO [115].

Therefore, the rapid responses and stringent adherence to NPIs to control the spread of the virus during the early COVID-19 pandemic, by the governments of these countries, was a necessary and precautionary reaction in this context. This also impacted on testing capacity, with the richer GCC countries like UAE and Bahrain leading in this area (with Jordan not far behind), compared to the developing North African countries, where adequate testing capacity was more of a challenge [113].

Thus, overall, the initial responses in the early COVID-19 pandemic in the MENA countries, though not optimally coordinated [9], were essentially still very effective out of pure necessity. This was despite wide disparities in the level of healthcare investment, various internal economic and political conflicts (e.g. Lebanon), and the ongoing related movement of refugees around conflict zones (e.g. Syria and Yemen).

### 2.4. Africa

**Government trust:** variable but generally low

**Testing capacity**: mostly poor, with limited capacity to expand

**Track/Isolation/Quarantine**: self-imposed and enforced due to limited hospital capacity

**Compliance with social distancing restrictions:** variable to high

**Masking:** variable—likely limited by available supplies

**Effectiveness of SARS-CoV-2 control:** variable to good—but limited testing capacity makes this difficult to assess accurately

Overall, healthcare resources are poor in Africa. For comparison, Uganda has 55 intensive care beds for a population of 42 million, compared to around 700 for a population of 10.4 million for Lombardy, Italy at the start of the pandemic. Other African countries like Mali, Burkina Faso and Liberia were even worse off, with just 20 ventilators a piece. In terms of hospital beds, there are approximately 1.2 beds per 1000 people across Africa, compared to 6.5 in France, 3.5 in Italy and 3 in Spain, USA and UK [116].

Yet, despite this, the COVID-19 case numbers and death toll in Africa has been surprisingly low—why? [117]. Some of this will be the result of under-testing and under-reporting, due widespread, and in many cases severe, national resource limitations. Yet, despite the poor healthcare infrastructure and funding, the mortality rate for COVID-19 in the continent in the early stages of the pandemic appeared to be less than those of Asia, Europe and North America [107,118,119]. Most sub-Saharan African countries score 2–3 on the WHO's CPRP scale [29]. Although there is a low rate of SARS-CoV-2 testing in many African countries, there are other possible reasons for Africa's low COVID-19 case numbers and deaths during the early part of the pandemic. These include a relatively younger population, a low population density, an outdoor lifestyle in a warm equatorial or mild temperate climate.

Many African countries imposed strict travel restrictions including border closures for several months like in Seychelles, Mauritius and Madagascar. What they lacked in hospital facilities, they made up in coordinated, rapid action in the community, based on decades of experience dealing with HIV and Ebola and other bacterial and parasitic diseases. These responses were government-led at national level, with strong support from the public, with good compliance with masking and social distancing measures—including staying at home where possible [107,119].

A report published by the Partnership for Evidence-Based Response to COVID-19 (PERC)—a public–private collaboration supporting evidence-based measures to reduce the impact of COVID-19 on African Union (AU) Member States—found that there was good compliance overall (at least 50%) with hand-washing, masking and social distancing measures during the first six months of the pandemic, including up to 85% compliance with mask use. This was based on a telephone poll of over 24 000 adults across 18 AU countries, conducted during 4–17 August 2020 [119].

The public health measures with the highest compliance (at least 75%) were those involving personal protection, such as hand-washing, avoidance of handshakes and other physical greetings, and masking. Those measures with the lowest compliance (around 50% only) were ones that impacted on food and economic security—and this was a common theme across various populations, globally, not just those in low and middle income countries (table 1).

**Table 1.** Survey of participants from African Union Member States, on questions of what public health and social measures they viewed as absolutely or mostly necessary (Support), and to what degree they complied with these measures. Adapted from graphic 8 [119].

activity	supported (%)		adherence (%)	
	absolutely	somewhat	complete	mostly
personal measures				
washing hands/using hand sanitizer	86	11	68	19
avoiding handshakes/physical greetings	77	16	58	18
wearing a face mask in public	84	12	71	14
public gathering measures				
avoiding places of worship (churches, mosques)	43	25	44	16
avoiding public gatherings and entertainment	68	20	57	18
measures restricting economic activity				
staying home	42	27	33	17
reducing trips to the market or store	53	29	38	23

Another interesting finding from this report was that while many Africans (around two-thirds overall) believed that COVID-19 would impact on the people in their population, only half of these (about one-third overall) believed that it would infect them directly (table 2). This belief makes it all the more remarkable that the compliance with most of the NPI measures was so high. In addition, within these surveyed countries, mask-wearing ranged from 48% (Tunisia) to 97% (South Africa), with 96% of respondents declaring that they had masks ready to wear, and that 85% of them had worn masks in the previous week [119].

**Table 2.** Survey of respondents from African Union Member States to two questions. Most people believed that COVID-19 would be a major problem for their country, but that their individual risk of catching COVID-19 was low. Adapted from graphic 10 [119].

African Union country	‘COVID-19 will affect very many people in my country’ (strongly or somewhat agree) (%)	‘personal risk of catching COVID-19’ (high or very high risk) (%)
All	68	29
Cameroon	53	24
Côte D'Ivoire	40	24
Democratic Republic of Congo	55	25
Egypt	64	27
Ethiopia	87	35
Ghana	59	24
Guinea	56	20
Kenya	78	31
Liberia	55	33
Mozambique	81	38
Nigeria	51	30
Senegal	80	32
South Africa	88	49
Sudan	85	22
Tunisia	62	17
Uganda	73	26
Zambia	76	34
Zimbabwe	65	24

In addition, the generally younger age of the population overall (only 3% of the population are over the age of 65 years), with very few elderly residential homes (as most people retired to their rural home villages that had very low population densities), helped to reduce the spread and impact of the virus [107].

Many Africans spend a lot of time outdoors, and this lifestyle, together with the hot dry weather reduces the airborne and surface survival of the virus, reducing further the risk of exposure and successful transmission. Spending time outdoors with skin exposure to sunlight also enhances their vitamin D production which is known to boost host immunity [120,121], which may possibly reduce the rate of successful infection and severe disease with COVID-19 [122,123]—as has been indicated more definitively for influenza and other respiratory viruses [124,125]. Others have hypothesized that the chronic exposure of the African population to many pathogens could have induced some tolerance to inflammation; together with the widespread use of live attenuated vaccines, like the Bacillus Calmette-Guérin (BCG) vaccine, which may elicit a bystander protective effect [126].

Another important factor that may have limited the impact of COVID-19 in Africa was that the level of connectivity between most of African countries and surrounding regions is generally relatively limited, and much lower than between other countries in other parts of the world [127]. Thus, any COVID-19 travel restrictions imposed across AU countries would have had a more substantial and quicker impact on limiting the spread of the virus across national borders—at least during the first pandemic wave. In some support of this, the AU countries that were the most affected during the first wave were those with higher connectivity to other continents, such as Morocco, Egypt, Nigeria and South Africa.

All of these factors, coupled with their experience, knowledge and application of traditional public health measures—tracing and isolation of confirmed infected patients and the quarantining of their contacts pending test results—managed to control the virus fairly effectively during the first pandemic wave (March–June 2020), whilst it was devastating countries in North and South America, and Western Europe. However, indirect impacts of COVID-19 may result in rises in the numbers of HIV, tuberculosis and malaria infections as people are unable to be tested and/or receive therapy during the pandemic-related restrictions [128].

Some African counties have suffered massively economically as a consequence of these restrictive measures, such as South Africa where 2.2 million jobs were lost during the first half of 2020 as a result of their stringent lockdown measures [107]. This will affect the degree of compliance with social distancing measures as most African economies are based around informal, casual work that cannot be performed, socially-distanced, remotely from home. The COVID-19 pandemic has further exaggerated pre-existing social and economic, as well as healthcare inequalities across the continent, with much of Africa's aid, supply chains, trade and tourism income, normally coming from North America, Europe and Asia, being severely curtailed during the pandemic [116].

We also know that the inadequate testing can lead to a gross underestimate of COVID-19 cases and deaths, with one longitudinal post-mortem survey from Lusaka, Zambia finding that one in five deaths were SARS-CoV-2 PCR positive—almost none of which had been screened for COVID-19 ante-mortem [129]. Further seroprevalence studies conducted in several countries have demonstrated that official numbers based on testing largely underestimated the spread of the epidemic, with one more realistic estimate of 450 000 COVID-19 cases far greater than the 5000 cases officially reported in Zambia [130]. Similarly, in Madagascar, a nationwide seroprevalence study in blood donors found far higher seropositivity rates for SARS-CoV-2 IgG than would have been predicted from the PCR-confirmed case numbers officially reported [131].

Another contributing factor to the underestimates of African COVID-19 incidence can be explained by long-standing population behaviours. Even when healthcare and SARS-CoV-2 testing is accessible, people may not come forward to be tested if they have mild and/or non-specific symptoms, particularly when costs are involved and/or the healthcare centre is some distance away. For example, 22.8% of patients in Kenya who were eligible for healthcare services declared not seeking healthcare even when being ill, with 44% citing cost and 18% travel distance issues [132].

Also, the scarcity of reagents and laboratory testing capacity, often meant that the contacts of COVID-19 cases were not systematically tested. Finally, the fear of stigmatization, or the impact of mandatory confinement on food and economic security for people testing positive, may have made some reluctant to be tested, again reducing the overall reported incidence of the virus in AU countries [133].

These relatively low COVID-19 figures across Africa during the early stages of the COVID-19 pandemic [134], are all the more remarkable when the wider context is considered. Although the population is younger overall, there are other serious endemic diseases and conditions present that impact on underlying population health, including malnutrition, tuberculosis, malaria, HIV/AIDS, Ebola, Lassa fever and various parasitic infections, as well as a lack of consistent access to clean water and food in some regions, and ongoing civil conflicts in others—some of which make COVID-19-related issues one of their lowest priorities.

### 2.5. Western Europe

**Government trust:** moderate to high

**Testing capacity:** initially limited, but rapidly increased

**Track/Isolation/Quarantine:** initially poor, but improved with variable degrees of compliance

**Compliance with social distancing restrictions:** initially good—though constantly challenged

**Masking:** not initially—but rose dramatically in some of the worst-affected countries later

**Effectiveness of SARS-CoV-2 control:** generally poor to moderate

Most Western and Central/East European countries score 4–5 on the WHO's CPRP scale, though the Ukraine only scores 2 [29]. Every health resource at their disposal was required to manage the first pandemic wave of COVID-19 in those countries. Similar to the response in the USA that of Western Europe was one of ‘wait and see’ and of essentially downplaying the risk from the virus—hoping that it would not extend beyond East/Southeast Asia—as was seen for the SARS-CoV-1 outbreaks of 2003 [135].

This attitude persisted for one to two months even after cases were being imported into various different Western European countries in late January 2020, particularly from skiers returning from their winter holidays [136], after mingling with skiers at ski resorts from East/Southeast Asia who were carrying the virus. Even after these early cases were identified, some large-scale sports and other social events continued to take place in various countries around the world, including in the UK and Australia [137,138]. Recent revelations about a ‘lost’ coronavirus pandemic plan from the UK, make the subsequent pandemic-related chaotic and disorganized responses seem all the more poignant, in that many COVID-19 cases and deaths could have been avoided if this plan had been available and closely followed [34].

More concerning were the efforts to downplay the risks of infection from airborne virus [139], and from individuals with asymptomatic infections who were less likely to have been tested but who were able to carry and transmit the virus [140,141], as well as any risks of virus infection in and transmission from children [142–146]. The contrasting response from most Central/East European countries was sufficiently different to merit a separate section (see below).

This reluctance to perceive the virus as anything more than an ‘Asian flu’ that would stay in that region and eventually die out persisted for precious weeks, with some Western European countries authorizing the delivery of large quantities of PPE (masks, gloves, gowns) to China—rather than increasing their own stockpiles. This was clearly done on the grounds that these Western countries did not consider SARS-CoV-2 to be a threat to their own populations or healthcare workers—despite COVID-19 cases already beginning to appear and spread in the UK and multiple EU countries [147,148].

In the UK, it was noted by some that an attitude of ‘British exceptionalism’ seemed to hampering the UK's response to the pandemic, with the belief that the UK would not be badly affected by the pandemic—then even when it was, refusing to learn from East/Southeast Asian countries that had managed the pandemic well [149–152]. The attitude of other Western European governments at this time was similar, even after the WHO's earlier declaration that the novel coronavirus constituted a PHEIC on 30 January 2020 [1] then later, a pandemic on 11 March 2020 [2].

Subsequent to this, there was a lot of confusion across Europe (as well as the rest of the world) about the evidence for the comparative importance of the different routes of transmission of the virus, i.e. contact versus large droplet versus aerosol transmission and combinations of these [153–156]; the effectiveness of masks (or the clumsier term ‘face coverings’) [157,158]; the role of asymptomatic infection and transmission and the importance of screening for such ‘silent’ COVID-19 cases [159–163].

Many media and academic articles around this time also started to note that Western European countries seemed to be unable or unwilling to learn from countries (including some former colonies) in East/Southeast Asia, who were controlling the virus extremely effectively [38,164–169], where the pandemic had been managed much better in terms of case numbers and deaths. This had been achieved in East/Southeast Asian countries by early, rapid and dramatic NPI responses in terms of closing borders, locking down cities, maintaining social distancing and universal masking. Similar responses were adopted early on in the pandemic by their much closer Scandinavian and Central/Eastern European neighbours, who also had achieved good control of the virus during the early pandemic (March–June 2021) [170].

Most of the Scandinavian countries (Norway, Finland, Denmark, Iceland) generally managed to control the spread of SARS-CoV-2 very effectively compared to their Western European counterparts, with fewer than 500 deaths per million population [4]. Unlike their Western European neighbours, these countries took the threat seriously and reacted quickly to reports of the evolving pandemic in China, instigating social distancing and national lockdown measures promptly, including the closing of schools, social gatherings and non-essential businesses [171–174]. They also had several advantages over some of the worst-affected, larger Western European countries, including excellent and well-resourced healthcare systems, good internet connectivity, small populations (~10 million or fewer) with low population densities, together with a culture of high compliance with government guidance and high levels of trust in their countries' leadership [173,174].

Other cultural differences that helped to reduce the spread of the virus amongst these populations was a general preference to stay at home, avoiding social gatherings, being happy to be alone, with a normally ‘hands-off’ approach to social interactions, i.e. little hand-shaking, hugging or kissing upon greeting one another [173,174], which is more prevalent in the cultures of some of the hardest hit—and less compliant—Mediterranean European countries (Italy, France, Spain, Portugal) [174].

Scandinavian countries also invested heavily in testing, some more than others, with Iceland eventually performing around 3.1 million tests per million population, and Denmark a massive 14.5 million tests per million population (both as of 7 October 2021), compared to around 1.1–1.5 million tests per million population for Finland, Norway and Sweden [4].

Although Sweden took a different path in their COVID-19 pandemic response, their eventual numbers of COVID-19 deaths per million population (1460 as of 7 October 2021), were still lower than the larger Western European nations—except for Germany (1123 as of 7 October 2021).

There has been much criticism of the more relaxed approach in the Swedish pandemic response, which was perceived as a natural ‘herd immunity’ strategy [175,176]. Whilst this has led to a much higher COVID-19 case fatality rate than the other Scandinavian countries that were perceived to have much more stringent pandemic restrictions in place, one analysis suggests that Finland and Norway had even less stringent pandemic controls in place for most of the pandemic with far fewer deaths [176].

Differences in the way Sweden is governed may account for the dominant role of the Swedish Public Health Agency and its famous state epidemiologist, Anders Tegnell, in determining their response to the pandemic. In contrast, Denmark and the other Scandinavian countries are predominantly governed by political leaders and their ministries, who may be more likely to follow the trend in applying pandemic restrictions seen in other European countries [177].

Masking generally remained low (below 10%) in Scandinavian countries throughout the first wave, but increased steadily in other Western European countries, e.g. from 0% to 30–40% in the UK, and rose dramatically to 80–90% in some of the hardest hit countries (France, Spain, Italy) within one to two months, with Germany later following suit, where masking reached over 60% [158].

One aspect that was dealt with relatively well by the Western European nations, and which compared favourably to other developed non-EU countries elsewhere, was the financial support for workers (both employed and self-employed people) and businesses, though the degree and nature of this support varied considerably across the EU.

For example, the UK, Denmark, France, Germany and Sweden only compensated employees for hours that were no longer worked (capped by either a fraction of their total wages or a maximum payment limit). Elsewhere (Australia, New Zealand, Ireland, Canada and the USA), all employees were given a wage subsidy if their businesses had suffered a major loss of turnover (ranging over 15–50%) during the pandemic. These wage compensation schemes (known as ‘furlough’ in the UK) also extended to the unemployed. In the UK, Ireland and Australia, a means-tested flat rate of unemployment benefit was paid out, regardless of any previous wages. This was in contrast to most other countries where eligibility for any unemployment benefit was linked to previous earnings and required claimants to have a record of previous employment and any unemployment insurance scheme for a minimum period. For the self-employed, this is where there was most variation, with the UK, France, Denmark and the US creating special schemes for this purpose. The UK, France and Denmark compensated the self-employed with up to 80% of past profits, and the USA, along with Sweden, paid out up to 70–80% of past profits based on new unemployment assistance (US) or pre-existing unemployment insurance (Sweden) schemes. Germany took a different approach, offering the self-employed a business grant to cover their fixed costs for three months up to a total of 9000 euros [178].

Although most Western European countries had a pandemic influenza plan, they had little or no experience with rapidly spreading zoonotic pathogens, such as SARS-CoV-1, MERS-CoV, or any human outbreaks of avian influenza in their local populations, which likely made them somewhat complacent. Interestingly, many Western European researchers wrote many academic articles describing and analysing outbreaks of these viruses (and others, like Zika virus in Brazil and Ebola virus in West Africa) elsewhere, but somewhat surprisingly, very little, if any of this expertise translated into government policies urging early and comprehensive actions when the virus entered and spread amongst their own populations—as starkly demonstrated by the surging COVID-19 cases in the first waves in Western Europe [179,180].

A lot of research effort and funding seemed to go into repeating studies that had already been performed previously for the 2003 SARS-CoV-1 outbreaks and the 2009 influenza A(H1N1)pdm09 pandemic. Many aerosol and airflow visualization studies were performed during the COVID-19 pandemic that were actually similar to or repeats of previous studies published 5–10 years earlier, on topics such as the effectiveness of masks to contain outgoing aerosols [181–187] and real-time, non-invasive airflow visualizations produced by human volunteer respiratory activities, like talking breathing, shouting, singing [188–191]; revisiting the issue around aerosol-generating procedures (AGPs) that were found to be a risk for the earlier 2003 SARS-CoV-1 outbreaks [192], though less so with the 2009 pandemic influenza A(H1N1)pdm09 virus [193], and SARS-CoV-2 [194].

Part of the confusion and ineffective control of the virus in the early pandemic also arose from the contrasting opinions of different scientists, with some advocating some degree of natural ‘herd immunity’, i.e. allowing the virus to spread naturally without the use of national lockdowns [195]; compared to a more ‘zero COVID’, ‘total lockdown’ approach, as demonstrated earlier by China in Wuhan [196]. Notably, signatories to the ‘Great Barrington Declaration’ opposed this national lockdown approach in favour of a more ‘focused protection’ strategy, where only the vulnerable would have to shield—though the practical details of exactly how this selective shielding strategy would be implemented were never explained [197].

The other factor that led to a subsequent second wave was the impatience to open up the economy and international travel after the first COVID-19 wave in June 2020, as COVID-19 cases eventually dropped to very low levels after the first wave lockdown. This ‘lockdown–relaxation–lockdown’ pattern was repeated across many Western European countries due to the competing pressures from health, economic and education lobbies, together with some selective interpretation of the data. For example, analyses at the time were interpreted as saying that there were few transmissions arising in the hospitality sector [198], which was contradicted by multiple reports from elsewhere [199–201], which tallied more closely with what we knew about how this virus mostly transmits between people, i.e. via aerosols over short, conversational distances, indoors, which occurs in all indoor hospitality scenarios.

All of this mixed and confused messaging, together with the rapid instigation then reversal of pandemic restrictions in the summer of 2020, unfortunately, set the scene for an even bigger second COVID-19 wave across Europe in the autumn/winter of 2020 [202–205].

### 2.6. Eastern Europe

**Government trust:** low to moderate

**Testing capacity:** initially limited, but gradually increased—availability of PCR kits was an ongoing problem and limited testing capacity

**Track/Isolation/Quarantine:** quite successful due to early responses with few COVID-19 cases

**Compliance with social distancing restrictions:** enacted early, with mostly good initial compliance

**Masking:** required in most countries

**Effectiveness of SARS-CoV-2 control:** generally good during the early pandemic (first wave)

Like some of the MENA and many African countries, most Central/Eastern countries (Hungary, Slovakia, the Czech Republic, Romania, Poland, Bulgaria and Ukraine) went into some form of national lockdown much earlier in March 2020, as they saw the virus spreading in Italy and the potential impact of COVID-19 cases on their healthcare system and the economy. This rapid response was likely due in part to an awareness of the fragility of their own healthcare systems, which may not have coped with such massive surges in COVID-19 related admissions, which had brought the Italian health services close to collapse [206].

A comparison of four Central/Eastern countries (Hungary, Poland, Lithuania and Slovakia) found that they all responded promptly with social distancing measures being imposed during 11–14 March 2020. This came with support for employees and the self-employed with flexible working conditions; businesses in terms of financial support, tax breaks and subsidies; and families in terms of paid sick leave for parents, extended maternity leave as needed and the suspension of mortgage payments for those unable to pay. As with the Western European countries and elsewhere, there was also a limited form of financial support for the unemployed, with each country offering different amounts under different schemes—the generosity of which was often linked to the prevailing political situation at the time. Lithuania offered the most generous social support packages of these four countries—but its president was also facing parliamentary elections in Autumn 2020, so policies that were popular would bring support from voters [170].

Despite differing approaches to worker compensation and social assistance, other Central/Eastern countries like Romania, the Czech Republic and Bulgaria also followed the same pattern, with an early rapid response to the pandemic and good control of the virus during the early stages.

Masking was variable across the Central/Eastern nations, with some like the Czech Republic, Slovakia, Poland, Bulgaria, Bosnia and Herzegovina making mask-wearing mandatory as early as March 2020 [158,207], and others like Romania resisting the wearing of masks [208]. This was on the backdrop of other issues such as inadequate medical staffing, with some doctors leaving for better paid and better equipped jobs elsewhere, such as those from Romania [208] and Hungary [209]; faulty equipment, including Russian ventilators used in Belarus that malfunctioned and caught fire killing some patients; doctors who had to work without adequate PPE [210]; and insufficient laboratory testing capacity, e.g. in Hungary, either due to lack of SARS-CoV-2 PCR kits and/or finance, as a result of poor planning [209].

In this context, it is notable that the trajectory of two other Central/Eastern countries, Russia and Belarus, more closely followed that of some Western European countries with relatively high COVID-19 case numbers during the first wave. As elsewhere, this was mainly due to a lack of early and adequate responses by these countries, with Russia's reaction being delayed and less coordinated [211], but Belarus's being more of a refusal to acknowledge the severity of the pandemic and take appropriate early actions [210].

One problem that Central/Eastern countries share with Asia is migrant workers. Many Central/Eastern citizens work in the wealthier Western European countries in the hospitality, healthcare, agriculture and food production, and construction industries, from where their return during the early part of the pandemic brought more virus back into their home countries [212]. This was a problem particularly for Romania, which has over 3 million of its citizens living abroad [208,213].

As described for some Asian countries above, it is important that such migrant workers are given careful consideration. If they are simply asked to return home, they risk carrying the virus back with them to their home countries; if they are to remain in the country of their employment, then they need to be compensated and cared for like the local citizens—otherwise, the virus will spread rapidly amongst them, if housed in cramped impoverished conditions, to then act as a virus source for the local population.

### 2.7. North America (USA, Canada)

**Government trust:** low to moderate

**Testing capacity**: initially limited, but rapidly increased

**Track/Isolation/Quarantine**: initially poor, but improved with variable degrees of compliance

**Compliance with social distancing restrictions:** variable to moderate—but constantly challenged

**Masking:** not initially—much resistance and ongoing debate about effectiveness

**Effectiveness of SARS-CoV-2 control:** poor to moderate

Both Canada and the USA score a maximum of 5 on the WHO's CPRP scale [29], which makes it surprising that the COVID-19 pandemic has hit these North American countries so hard. Despite Canada's previous experience of the SARS outbreaks of 2003, its early response to the COVID-19 pandemic was relatively slow and insufficient.

The main failings of the Canadian COVID-19 response was a lack of overall capacity in terms of testing and PPE supplies—as well as a slow response and failure to apply the ‘precautionary principle’. Examples of this include the Public Health Agency of Canada (PHAC)'s reluctance to accept the risks of asymptomatic transmission and the possibility of airborne spread of the virus. There was also hesitation to close international borders, a reluctance to accept that the virus might be airborne, as well as recommending the use of masks in public. The inadequate supply of PPE was further exacerbated by the destruction of millions of expired N95 masks and the sending of 16 tonnes of PPE to China early in the pandemic. These failures led Canada to have more COVID-19 deaths than Taiwan, Hong Kong and China combined. During the 2003 SARS outbreaks, Canada had the highest numbers of cases and deaths outside of Asia, with over 400 infected and 44 deaths, and one could argue that despite the additional measures implemented since then, it still had not fared much better during the early days of the COVID-19 pandemic [214].

Although 80% of the measures recommended by a local epidemiological report (led by David Naylor) written after the 2003 SARS outbreaks had been implemented at one of the hardest hit hospitals in Toronto (Sunnybrook Hospital), these were not enough to contain the COVID-19 pandemic. These measures included: the presence of a pandemic plan, improved communications between hospital, public health and government officials, infection control staffing, airborne isolation room capacity, syndromic triage and surveillance, diagnostic testing capacity, and supplies of PPE with fit-testing [215].

Canada has been particularly resistant to the concept of aerosol transmission for SARS-CoV-2 since the beginning of the pandemic [216]. Part of this may have been due to an early lack of PPE, including N95 masks (used for aerosol exposure)—exacerbated by the earlier donation of 16 tons of PPE to China from their National Emergency Strategic Stockpile (NESS) [214].

Finally, any changes in the public health messaging were made more difficult by the Canadian federal system, where health is a provincial jurisdiction. This meant that, in the case of PHAC eventually recognizing the potential for airborne transmission of SARS-CoV-2, for example, the same message would have to then filter down through 10 provincial and 3 territorial governments for appropriate aerosol infection control measures to be adopted more locally.

In the USA, the COVID-19 pandemic began during the Trump administration. The initial warnings about a potential pandemic due to a novel coronavirus arising in China were met with skepticism and some derision by the US presidency, with the virus being readily played down or dismissed as just a ‘flu’ that will have passed by Easter (2020) [217,218], and this attitude was adopted by some of the population. Little or no additional preparedness was taken to combat the emerging virus, though there was substantial coverage in the media of the evolving situation in China—including the building of a new hospital in China within 10 days [219].

When the virus did eventually reach the USA, testing and reporting were inadequate. The CDC was subject to constant pressure and interference from White House officials with little or no public health expertise or experience, leading to rushed and flawed test design, and a confused and disorganized response during the early part of the pandemic [17]. Each state had its own surveillance and reporting system, often outdated and underfunded [220], making it challenging to obtain an accurate picture of the nationwide spread of the virus in a timely manner.

The USA's response was characterized by a lot of mixed and confusing messaging, particularly around masks and their effectiveness. At one point, Dr Jerome Adams, the US Surgeon-General, tweeted ‘Stop buying masks!’ then going on to say that they do not prevent the general public from catching coronavirus, despite the fact that healthcare workers were wearing them in hospitals for this reason [20].

Even after masks were finally recommended as a protective intervention, many Americans saw this as an infringement of their civil liberties, with numerous high profile demonstrations to protest against this mandate. Indeed, historical evidence recorded that this rebellion against mask-wearing was also evident during the 1918 ‘Spanish’ flu pandemic, with one article noting that America was built on a culture of rebellion [221]. This can be contrasted against the more compliant and voluntary mask-wearing culture in East/Southeast Asian populations, which contributed to their very effective virus control [222].

Also, as the pandemic response rapidly became highly politicized [223], masking in particular was seen more as a sign of support for the Democrats than the Republicans. In addition, misinformation and conspiracy theories abounded about the origins of the virus, treatments for the disease, vaccine efficacy and safety [224], and whether the entire pandemic was a hoax [225]. This all detracted from the real issues around implementing and following public health guidance to control the spread of the virus. Such delays in implementing adequate social distancing measures potentially resulted in large numbers (at least 20 000–40 000) of unnecessary and preventable deaths, as inferred by several models, not just in the USA, but also in the UK [226–228].

On the specific subject of aerosol transmission, several reports commented on the reluctance of key players amongst the national infection control policy-makers and advisors to accept this route of transmission—even going as far as excluding relevant literature from ‘evidence-based’ reviews to inform policy—particularly in the USA, UK and Canada, where measures to control airborne transmission were strongly resisted in the early part of the pandemic [139,216].

Later, as the pandemic spread across the USA, in some states like California and Ohio, the public were more understanding and compliant with the banning of large gatherings, including the closure of various businesses that would encourage social mixing, like bars, restaurants and night clubs [229,230]; whereas in Texas, although there was initial compliance, when the prolonged closures threatened to put people out of business, some reopened in protest [231]. Thus, differences in public health guidance across different US states contributed to the general public's confusion.

Hence, the deficiencies in the responses to the early pandemic in North America can be summarized as: (i) downplaying the seriousness of the pandemic and side-lining experts who advised otherwise. This led to delays in an adequate pandemic response resulting in the rapid spread of the virus; (ii) slow and flawed testing. This led to delays in detecting and recognizing then reacting promptly to the rapid spread of the virus with appropriate public health interventions; (iii) inadequate tracing, isolation and quarantine of those infected or exposed. Without any means of enforcing such measures (which would almost certainly be met with protests against the violation of civil liberties), this led to onward spread of the virus throughout the population; (iv) confusing mask guidance. This was similar in many Western countries, where there were ongoing debates about the effectiveness of masks and exactly what type of evidence would suffice to settle such debates; v) airborne spread and hygiene theatre. The lack of recognition of aerosols as the main route of transmission for SARS-CoV-2 led to the ongoing ‘hygiene theatre’ of hand-washing—which clearly failed to control the spread of the virus to any degree [217,232].

The last aspect, ‘hygiene theatre’ (i.e. excessive hand-washing and surface cleaning), in particular, was common in many Western countries during the early part of the pandemic, and has been ranked as one of the least effective infection control measures for SARS-CoV-2 [25]. Again, this and other delays in implementing other more effective control measures likely led to many unnecessary and preventable COVID-19-related deaths [226–228].

As with other Western European countries, the USA adopted too much of a ‘wait-and-see’ attitude, reacting too late and not appropriately or comprehensively enough to curb the spread of the virus in the early pandemic, which made it much harder to control later on. These delays were due to various experts and government officials debating the evidence and its interpretation for the effectiveness of various public health interventions, including masking, whether the virus was airborne, whether asymptomatic cases could transmit the virus, as well as struggling to expand their testing, tracing, isolation and quarantine capabilities—and the related legislation—some of which were met with rapid resistance from politicians and the public alike. The precautionary principle to infection control states that early, pre-emptive action should be taken to protect life—even before a complete evidence base is gathered for such actions [214]. Many Western countries, unfortunately, failed to do this during the early stages of the COVID-19 pandemic, resulting in widespread transmission and seeding of the virus in their populations, resulting in some of the highest COVID-19 case numbers and deaths, globally.

### 2.8. Central and South (Latin) America

**Government trust:** low to moderate

**Testing capacity:** mostly poor, with limited capacity to expand

**Track/Isolation/Quarantine:** variable depending on local resources and support—but generally low hospital capacity

**Compliance with social distancing restrictions:** variable depending on guidance and local support

**Masking:** variable depending on guidance and availability

**Effectiveness of SARS-CoV-2 control:** poor to moderate

Like most countries in Central/Eastern Europe, Africa and MENA, the initial success of many Latin American (LA) countries (apart from Brazil and Peru) in controlling the spread of the virus during the first pandemic wave was due to the early and rapid implementation of control measures. Most LA responded promptly to the pandemic declared by WHO by implementing procedures to suppress transmission including lockdown, social distancing, use of masks and COVID-19 contact tracing. While these measures were effective in delaying the viral transmission in the community in the early phase, sustaining these interventions were difficult for multiple reasons.

A comprehensive view of the pre-pandemic Latin America healthcare situation at the onset of the pandemic found that these countries, in general, had a low total health expenditure of 5% to 9%, with the majority being less than 6%. Non-communicable diseases were the main cause of death and formed the major healthcare burden. Furthermore, healthcare delivery was found to be inefficient and the policies to improve performance and efficiency of the health system were usually adopted very slowly [233]. Therefore, when the COVID-19 pandemic arrived in LA, these countries already had healthcare systems that were struggling with their daily workloads.

Another study examined the potential causes of the uneven impact of COVID-19 and found that many of these were already present in LA before the start of the pandemic—which were also common in many other countries globally. These included the suboptimal availability and quality of healthcare, the widespread existence of co-morbidities in the community population, and suboptimal pandemic responses by individual national governments. These factors increased the difficulties of trying to control the virus in the early pandemic, as well as the later mitigation of virus transmission in the wider community [234]. This resulted in LA being one of the worst affected regions, globally, particularly for some individual countries like Peru, which reached the highest death rate in the world (6114.93 deaths/1 million population) [4,235]. Other long-standing problems in LA, such as political instability, corruption, social unrest, fragile healthcare systems and most importantly, widespread inequalities in incomes, healthcare and education, substantially worsened the impact of COVID [236].

As a further illustration of the relative fragility of healthcare systems in LA, most countries in Central and South America (Brazil, Chile, Costa Rica, Mexico, Argentina, Colombia, Ecuador, Panama, Mexico, Peru) score 3–4 (out of 5), with a few scoring 2 (Bolivia, Guatemala, Honduras, Paraguay, Venezuela, Nicaragua) on the WHO's CPRP scale [29]. In addition, an OECD review in 2020 noted that most of these countries had fewer healthcare professionals and fewer beds per 1000 population, with countries like Mexico, Costa Rica, El Salvador, Nicaragua, Honduras, Guatemala, Ecuador, Peru, Venezuela, Bolivia, Paraguay, Colombia, Chile and Brazil having 2.1 beds per 1000 or less, compared to the OECD average of 4.7. This healthcare capacity was also limited when it came to COVID-19 testing, with some countries, like Costa Rica, declaring early in March 2020 that suspected COVID-19 cases would not be reported as there was no capacity to test them. This chronic lack of healthcare funding also meant that these countries had weaker disease surveillance systems and public health capacity to test, track, and, respectively, isolate or quarantine those infected or exposed [237].

One of the most notable themes of the Latin America countries' response to COVID-19 was a distrust of their government and a generally poorly equipped and underfunded healthcare infrastructure. For example, in Brazil's early response to the pandemic that led to soaring COVID-19 case numbers and deaths, the national leadership, persistently refused to acknowledge the seriousness of the pandemic, characterizing COVID-19 as a ‘little flu’, and not following the science to mandate the wearing of masks or the need for social distancing—which just allowed the P1 variant to spread further and faster in an already crippled healthcare system [238–242]. At one point, Brazil's pandemic response was referred to as the worst in the world by Médecins Sans Frontières [243].

Some drugs were also widely recommended which had not yet shown any definitive benefit—though possibly some potential harm due to adverse effects—like chloroquine and ivermectin [244–246].

About 3 out of 4 LA citizens have little or no confidence in their governments, with 80% of them believing that there is widespread corruption [237]. This is not new, with the COVID-19 pandemic further revealing serious inequalities across these populations, not just in healthcare, but also the vulnerability of the largely informal workforce to economic hardship during social distancing measures. This, together with a mistrust of government advice and lack of financial support, meant that people still went out to work, allowing the virus to spread [247]. A consequence of this was the spread of COVID-19-related advice on both the conventional (television) and social (mobile phone) media platforms, as illustrated by a survey of populations across three LA countries (Colombia, Mexico and Venezuela). This revealed that people were adopting COVID-19 social distancing advice from international sources, often before they were mandated by their own governments. Such sources included the WHO, whose advice eventually manifested in their communities in several ways, e.g. maintaining social distancing whilst queuing (Bogata, Colombia), restricting restaurants to a take-out only service (Mexico City, Mexico), and restricting customer numbers in a market (Caracas, Venezuela) [248].

This also manifested to some extent in the adoption of masking before some countries' local government guidance recommended it, resulting in a diverse approach to this intervention. Masking was eventually mandated in: Chile, Dominican Republic, El Salvador, Guatemala (April 2020, in public places); Uruguay (May 2020, on public transport); Panama (June 2020, in public places); Brazil (August 2020, in schools, churches and stores) [237,249,250].

Although the timing, duration and degree to which these interventions were implemented varied considerably, using Chile's experience as an example, the overall responses to the early pandemic in LA (and indeed other) countries can be summarized as follows: (i) an increase in testing capacity and surveillance, explaining to the public the need for social distancing, masking, curfews and measures for isolation and quarantine—to attempt to detect, monitor and control the numbers of COVID-19 cases; (ii) the closure of educational institutions, businesses and other forms of mass gatherings, with the enforcement of isolation and quarantine—to further reduce the ongoing spread of the virus; (iii) an escalation to the lockdown of entire cities or regions—to reduce the reproductive number (*R*_0_) if the earlier smaller-scale measures failed to curb the spread of the virus [251].

### 2.9. Antarctica

**(based mostly on the experience of the British Antarctic Survey team)**

**Government trust:** Not applicable—see main text below

**Testing capacity**: Variable and dependent on the resources made available through the National Antarctic Programmes (NAPs) of the individual bases

**Track/Isolation/Quarantine:** Most NAPs operated a quarantine process prior to person's entering their respective Antarctic base, with some variation between nations as to duration and testing regime

**Compliance with social distancing restrictions:** No changes to base or ship life in ‘clean’ post-quarantine cohorts, with protocols in place to enforce bubbles within workgroups and living quarters, stagger mealtimes and distribute PPE if any generalized outbreaks occurred

**Masking:** none inside the bases in ‘clean’ post-quarantine cohorts

**Effectiveness of SARS-CoV-2 control:** excellent overall

During the early part of the pandemic, the Antarctic remained COVID-19-free due to stringent measures put in place by the scientists travelling and working there [252]. Unlike the other countries and continents described, Antarctica's only indigenous life-forms are non-human, and the human presence there consists mainly of researchers, support personnel and visiting tourists. Apart from the individual international bases (which are relatively isolated) and whatever local support infrastructure exists to service them, there are no other large, interconnected sites (e.g. schools, universities, hospitals, entertainment and sports venues, public transport networks, etc.) where large numbers of people can meet and socialize. This makes the transmission of the virus between bases much more difficult and more limited, even if it did occur. However, if there was an outbreak of COVID-19 at any of the individual international bases, there are very few with intensive care facilities and the required logistics to care for those who become severely ill.

Antarctic tourist cruises remain a vector risk for the importation of the virus. One report described a largely asymptomatic outbreak of COVID-19 on a ship that left Ushuaia, Argentina in mid-March 2020 (after the WHO's declaration of the COVID-19 pandemic), on a 21-day cruise of the Antarctic Peninsula via the Drake Passage (Danco Island, Paradise Bay, Lemair's Passage and Deception Island) that included Elephant and South Georgia Islands [253]. However, on Day 3, it was decided to cut the cruise to just 14 days (abandoning the South Georgia leg), due to various international border control and travel restrictions resulting from the declaration of the pandemic. The 128 passengers and 95 crew were having routine temperature checks during this time. None of the passengers had previously transited through the most severely affected Asian countries (China, Macau, Hong Kong, Taiwan, Japan, South Korea) or Iran, in the three weeks prior to boarding. Yet on Day 8, the first passenger spiked a fever. This was followed by three crew becoming febrile on Day 10, then two passengers and one crew developing fever on Day 11 and then three more passengers on Day 12. Eventually, out of the 217 passengers and crew on board, 128 (59%) tested positive by the US CDC SARS-CoV-2 PCR test. Of these, only 24/128 were symptomatic with 104/128 (81%) being asymptomatic. Of the 24 symptomatic cases, 16 had fever and mild symptoms, but 8/128 (6.2%) needed medical evacuation, with 4/128 (3.1%) of these needing intubation and mechanical ventilation.

Clearly if a similar outbreak of COVID-19 occurred in any of the Antarctic bases during the winter ‘closed’ season (when transport in and out of Antarctica was not possible) this would have serious implications for those needing intensive care support [254,255].

Antarctica differs from the other regions discussed in this article in that it has no overall government. The individual bases are governed by their respective nations. Infection control and screening of personnel manning these bases for SARS-CoV-2 is thus the responsibility of individual National Antarctic Programmes (NAPs), rather than their national governments, directly. The NAPs are collectively part of the Council of Managers of National Antarctic Programs, which has a medical advisory body—the Joint Expert Group of Human Biology and Medicine (JEGHBM) which includes medical representation from many Antarctic Programs. JEGHBM have issued policy and guidance documents that have recommended best practice guidelines with the aim of maintaining the Antarctic continent as COVID-19 free as possible. This is critical due to the potential impact on remotely deployed personnel in a setting with limited medical facilities and long repatriation times—and to maintain biosecurity. Other commercial, mostly tourist, activity in Antarctica virtually ceased during the early phase of the pandemic, with policies and processes driven internally within each company.

Thus, in this context, during the early pandemic, enhanced screening of those people at increased risk of severe COVID-19 was used in some bases. SARS-CoV-2 PCR testing capability was introduced into ships and bases of several NAPS early in the pandemic.

For the British Antarctic Survey (BAS) bases, a strict pre-departure screening programme was put in place, consisting of a 14-day quarantine period with SARS-CoV-2 PCR testing on Days 1, 10 and 12 requiring to be negative before travel was permitted. This testing regimen was deemed to provide the highest level of reassurance that an uninfected and medically low risk cohort entered these remote bases. In addition, ship or air crews arriving in Antarctica to collect or deliver cargo had no contact with any BAS staff, but had to remain in their own isolation bubbles during any overnight stays on the continent. The BAS team also reduced their overall staffing levels from 500 to 180 during the COVID-19 pandemic period.

Close living environments and the interdependent nature of the deployed staff, in addition to the remote setting, enforced most of the protective measures at the entry phase into Antarctica. Many bases and ships do not have the ability to undertake full isolation and/or quarantine of infected personnel. Dedicated isolation facilities are often not routinely available and plans were based on *ad hoc* requirements. Visitors transiting through by plane or ship, delivering cargo or conducting other administrative duties, maintained strict social distancing and used PPE, with separate accommodation (if needed), and had no direct contact with base personnel. All cargo was left for 72 h and disinfected before being handled.

This Antarctic COVID-free status was eventually lost by December 2020 when 36 cases (26 Chilean army and 10 civilian maintenance personnel) of COVID-19 were identified at the Chilean Bernardo O'Higgins research station—one of 13 Chilean Antarctic bases [254].

Prior to this, at least 1000 personnel across 38 Antarctic stations had managed to stay COVID-19-free throughout the Antarctic winter [256]. Due to reduced staffing numbers and the relative isolation between the Antarctic bases, this outbreak was contained [255], though another 3 COVID-19 cases were detected on a Chilean navy support ship with a crew of 208, sailing in the area during November 27 and December 10 [254,255].

## 3. Summary and synthesis

These brief overviews of various countries and their reactions to the COVID-19 pandemic have revealed some useful themes to help us understand how and why some approaches worked better than others to control the spread of the virus in their populations—and reduce the numbers of COVID-19 cases and deaths.

The benefits of the quick and comprehensive reactions by Australasian, East/Southeast Asian, most East/Central European, Scandinavian, many African, most MENA, and some South American countries that did not initially downplay the potential seriousness of the novel coronavirus, during January–April 2020, was soon evident. Almost in parallel, the disastrous (in terms of COVID-19 case numbers and deaths) results of the slower ‘wait-and-see’ response of most Western European, North American and some South American countries, accompanied by government rhetoric downplaying the severity of the virus and any potential pandemic, was also plain to see.

Some of the tragic consequences of this confused and inaccurate interpretation of the early epidemiological data (including the ineffectiveness of the ‘hand’ hygiene theatre to control the spread of the virus) [232] and a lack of application of the precautionary principle (i.e. if you are not sure, protect against it) [234], likely led to avoidable deaths in bus drivers [257], choir members [258] and healthcare workers [259], who did not have the correct information nor the appropriate PPE for protection against an airborne virus in the early pandemic [260,261].

Given the differences in resource availability, competing priorities and government styles across different regions, it may not be possible or desirable to adopt a single universal global approach to the next pandemic—though perhaps a more flexible tiered approach may be applicable. Some of the following underlying principles upon which some countries based their pandemic responses may be considered for such a more tiered approach.

### 3.1. Experience of novel emerging infections

The countries that experienced SARS-CoV-1 in 2003 were mostly those in East/Southeast Asia and Canada, though there were a few cases identified in Europe, North America and Australasia [262]. Most of the affected East/Southeast Asian and Australasian countries used this experience to take a more precautionary and early response approach to implementing measures to control the spread of SARS-CoV-2 in their populations. North American and European countries that only had a few cases of SARS-CoV-1 failed to react in the same way and experienced severe COVID-19 outbreaks in their populations. The exception to this was Canada that actually experienced two large waves of SARS-CoV-1 in 2003 [263], but still reacted relatively slowly to the new SARS-CoV-2/COVID-19 pandemic threat.

Conversely, from the viewpoint of the Western countries, since SARS-CoV-1 in 2003 was mostly limited to East/Southeast Asian countries (with the exception of Canada), and eventually died out in response to the stringent public health measures implemented there [135], why would this not happen again? There was an initial general assumption in many Western countries that SARS-CoV-2 would behave just like SARS-CoV-1 and essentially not spread much beyond East/Southeast Asia so there was no need to worry.

Although one can understand why this line of reasoning seems valid, especially based on the decades of accumulated experience with the various influenza pandemics [264], where each virus behaved quite similarly to the previous strain despite the antigenic shift, the difference between SARS-CoV-1 and SARS-CoV-2 was much more significant. This was particularly in the timing and the manner of how it was transmitted. SARS-CoV-2 was soon found to transmit much earlier in the illness than SARS-CoV-1—and crucially even in those who were only mildly ill or even asymptomatic [265].

Worst still, these types of mild or asymptomatic SARS-CoV-2 infections made up the majority of COVID-19 cases, who remained in the community, quietly spreading the virus, as they were not sick enough to need hospitalization, when they would be isolated and tested. This revealed another flaw in the early testing strategies in Western countries.

Since they were less well-prepared than their East/Southeast Asian counterparts that had spent the years since SARS-CoV-1 2003 (and ongoing exposure to avian influenzas, e.g. A/H5N1, A/H7N9), in terms of expanding their public health surveillance, testing, tracing, isolation and quarantine capacity, the limited diagnostic testing and expansion capacity available in many Western countries was initially prioritized to screening those who were more severely ill and symptomatic. This left thousands, even millions of mildly or asymptomatic COVID-19 cases circulating, untested and undiagnosed, in the community to continue spreading the virus [266]. Inevitably, some of these asymptomatic or mildly infected individuals eventually had contact with elderly care homes directly or infected others who did, resulting in multiple fatal outbreaks [267].

Experience gives governments and public health teams more confidence to make quick decisions about the timing and severity of any interventions, as well as the need for their strict enforcement because they know the consequences of delayed implementation and inadequate compliance.

### 3.2. Confidence in their healthcare systems' ability to cope

For different countries, ‘over confidence’ or ‘lack of confidence in their healthcare systems' ability to cope with a surge of pandemic cases also determined their responses. More specifically, in most Western countries, there was some degree of over confidence in their healthcare systems and existing influenza pandemic plans, to be able to cope with any novel emerging pathogen—including another respiratory coronavirus. Early on in the pandemic, many Western countries considered SARS-CoV-2 to be just another ‘flu’, and they had successfully dealt with the 2009 A(H1N1)pdm09 pandemic—so why would SARS-CoV-2/COVID-19 be any different [135]?

Conversely, in many lower income Asian, Middle Eastern, African, Scandinavian and Central/Eastern European and South American countries, from the outset of the pandemic, they were concerned that their healthcare systems would not be able to cope with surges of novel coronavirus cases. This drove them to implement precautionary NPIs, such as social distancing, masking and travel restrictions very early on, to control the spread of the virus. This would give them more time to ramp up their testing capacity as well as prepare facilities and public health support teams for those needing to be isolated or quarantined.

Although both seasonal influenza (flu) and COVID-19 can present in similar ways (fever, cough, headache, fatigue, myalgia), with 50% or more of infections being mildly or asymptomatic and not requiring hospitalization, with similar modes of transmission (via both droplets and aerosols, as well as via direct contact and fomites), the pre-symptomatic period of virus shedding and transmission is longer for SARS-CoV-2 (~2 days) than for influenza (~1 day) [141,268].

Also, unlike influenza, most global populations have had no previous exposure or experience of SARS-CoV-2, so there was no pre-existing immunity to help mitigate the clinical disease severity. For those who survived SARS-CoV-1 in 2003, however, one study demonstrated the existence of cross-reactive, protective antibodies [269] that may mitigate COVID-19 severity. Yet for the vast majority of the global population, there was no such mitigating factor for more severe COVID-19, for which there was no specific treatment during the early part of the pandemic. Finally, long COVID syndrome does not have an equivalent syndrome in influenza, and other unusual COVID-19 complications, such as loss of taste and smell, tinnitus, and an increased risk of thrombosis, are not typically found with influenza. So there are significant clinical differences in the diseases caused by SARS-CoV-2 (COVID-19) and influenza that can differentiate between the two viruses [270–273].

It is known that infections with a high proportion of shedding and transmission before symptoms appear are very difficult to control [274]. Thus, the longer pre-symptomatic (2 days for SARS-CoV-2 versus 1 day for influenza) and longer duration of shedding post-symptom onset (10 days for SARS-CoV-2 versus 7 days for influenza) in COVID-19 patients [271], allows SARS-CoV-2 to spread earlier and for longer during clinical illness (or even asymptomatically) than influenza. This contributed to its overall higher R_0_ (basic reproductive number—the number of cases arising from a single infected case in an otherwise fully susceptible population), and rapid spread during the early pandemic [265].

Many respiratory viruses are now recognized to produce asymptomatic or mild symptoms that do not require hospitalization [275], but allow the virus to continue to spread, which also fit the pattern of infection for SARS-CoV-2 in the early reported case data from China [276–278].

Although it is unknown if the expert advisors in various countries were aware of these early Chinese case clusters, the precautionary approach by many of the lower income countries, due to very real concerns about the ability of their healthcare systems to cope, proved very successful in limiting the spread of the virus during the early pandemic period.

### 3.3. Competing priorities

Other considerations, were economic and social disruption—including large-scale closure events, such as schools, universities, hospitality, retail, travel and other customer-facing, non-essential industries. In particular, where the income in some sectors relied on the so-called ‘gig’ economy, where work was often piecemeal and cash-in-hand, and could not be conducted or protected easily if restrictions on social interactions were severe.

One of the main questions for all countries for the next pandemic is ‘when should we react?’ What indicators should we use to gauge when and how much to ‘lockdown’? As we have seen over the past 18 months during the COVID-19 pandemic, getting the timing and severity of this decision wrong can impact severely on the population in terms of social, psychological, educational and economic disruption.

Several industry sectors—such as hospitality and travel—are fragile and vulnerable to oscillations of 'lockdown then opening-up' cycles, so it is important to make the right decision at the right time—and this may explain the hesitation of many Western governments in implementing an earlier national level lockdown. Looking forward to future pandemics, this will always be potentially difficult at the beginning because each pathogen will have its own characteristics—but then a sensitive precautionary principle approach should then perhaps be the default option—as outlined in the Campbell report after Canada's experience with SARS-CoV-1 in 2003 [214,279,280]:

‘Where there is reasonable evidence of an impending threat to public harm, it is inappropriate to require proof of causation beyond a reasonable doubt before taking steps to avert the threat…that reasonable efforts to reduce risk need not await scientific proof.’

Various articles have offered some ideas for this, as well as using real-time tracking data to measure the impact of interventions as they are implemented [281–283], but ultimately, with any novel pathogen, there will be a lead time before we can optimize pandemic responses, during which, unfortunately, there will be severe illness and deaths.

Overall, the emergent theme from the countries that hesitated to put their populations into an earlier lockdown was one of trying to protect the economy, and to delay any social disruption (including in educational, retail, hospitality and entertainment venues, and travel restrictions), for as long as possible. However, the important insight that was missed (which comes with experience) is that unless the virus is well-controlled, the disruption to the economy and society will continue.

### 3.4. Inherent cultural differences

Our analysis shows that East Asia's success, compared with the six selected Western societies, can be attributed to stronger and more prompt government responses, as well as better civic cooperation. In particular, East Asian governments [that] implemented more stringent mobility control and physical distancing policies, as well as more comprehensive testing, tracing and isolation policies (except for Japan) since the early stages. The weaker policies in Japan are associated with the worst performance in containing COVID-19 among the five East Asian regions. [48]

Throughout the early part of the COVID-19 pandemic, multiple media articles highlighted the differing COVID-19 case numbers and deaths between Eastern and Western countries—along with the two ‘Western’ exceptions of Australia and New Zealand [40,166,167,222,284].

It was not only that East/Southeast Asian regions took the pandemic threat more seriously, earlier, and rapidly expanded their testing, isolation and quarantining capacity, together with more stringent, government-mandated restrictions. It was the fact that their populations were more compliant with the restrictions, despite the loss of individual freedoms, with a greater understanding and acceptance that this was for the greater good. Although stringent restrictions were also brought in for many Western countries, these were introduced too late, without enforcement, in populations that were less tolerant of, or compliant with them—which led to greater and more rapid spread of the virus.

One useful metric is available via Hofstede Insights [285], which offers an objective six-dimensional matrix by which different countries can be compared in terms of: *power distance*—the degree to which hierarchies in society are accepted (high score) or challenged (low score); *individualism*—whether a society tends to prioritize the individual needs (high score) over that of the group or population (low score = more ‘collectivistic’); *masculinity*—where society values winning and achievement (high score) above quality of life and caring for others (low score); *uncertainty avoidance*—where a society sticks to rigid rules and beliefs (high score) rather than deviating from the norm and taking risks (low score); *long-term orientation*—where a normative society seeks to preserve more links to the past and resists change (low score) over a more pragmatic, adaptive attitude to tackling future challenges (high score—more typical for many East/Southeast Asian countries); *indulgence*—the degree to which individuals in society feel able and willing to do what they want and follow their desires (high score—most Western countries) rather than act with restraint in the consideration of others (low score).

Of these six dimensions, the combination that might be most relevant to the successful, early control of a pandemic in a population could be: a high score in *power distance*—if the population accepts the government hierarchy and complies with pandemic restrictions willingly and comprehensively (which also requires a degree of government trust and a reliable and knowledgeable source of expert advice); a low score in *individualism*—people need to give up some of their civil liberties for the greater good of the population, to stop the virus spreading (as seen in Australia and New Zealand); a low score in *masculinity*—such that during a pandemic, it is not about an individual's winning or achievement, rather the need to preserve all life as best they can; perhaps an ambiguous score in *uncertainty avoidance*—as new, innovative ways to combat the pandemic may be required, if these measures are not already in place, e.g. for many Western countries that did not experience SARS 2003 or ongoing avian influenza exposure that may lack enhanced animal and human surveillance capabilities, and/or the accompanying expanded laboratory testing capacity that may have already been in place in East/Southeast Asian countries since SARS 2003; a high score in *long-term orientation*—indicating that populations are willing to drop past beliefs and traditions to adopt new ones as required to meet future challenges, e.g. like different pandemic pathogens; a low score in *indulgence*—as pandemic restrictions will require restraint from individuals (e.g. a reduction in social gatherings and other leisure activities) in a society that wants to successfully control the spread of the virus—for everyone's benefit.

**Figure 2.**
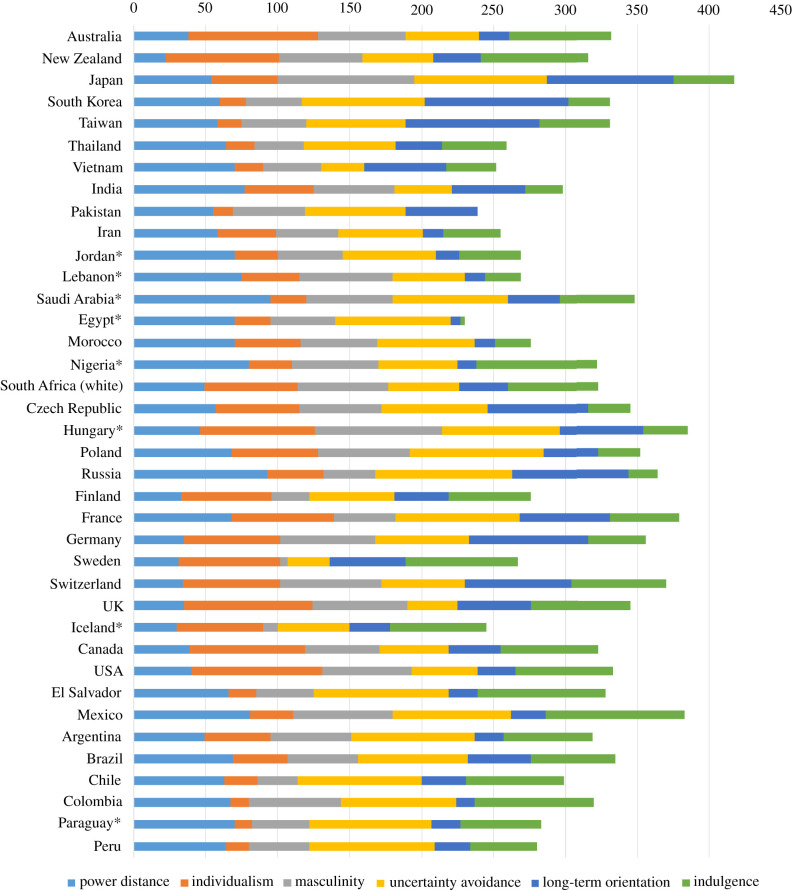
Examples of Hofstede Insights' six-dimensional comparisons of cultural trends across 38 countries arranged in geographical groupings following the order in the main text. Scores on different coloured bars range from 0 to 100. Asterisk indicates some parameters were estimated (https://www.hofstede-insights.com/country-comparison/argentina,brazil,chile,the-usa/) [285].

Figure 2 is based on scores generated by the Hofstede Insights website [285], and shows and compares populations at various positions in these spectrums, including the UK, USA, Peru and Brazil—which poorly controlled the spread of SARS-CoV-2 in the early pandemic—and South Korea, Vietnam, Taiwan and New Zealand, which controlled the virus much more effectively.

The comparisons between the Western nations, UK, USA and New Zealand show very similar scores for all of these dimensions, but New Zealand had the added advantage of an early, rapid, comprehensive response to the pandemic, with a high trust in their strong government leadership—enough to make most New Zealanders temporarily give up their usual attitudes in society within these dimensions. In contrast, populations in South Korea and Taiwan mostly scored at the opposite end of the spectrum for these dimensions—though Taiwan scored higher than perhaps expected, in the indulgence category compared to South Korea. Results from other selected countries from different continents are also shown.

Success or failure in managing a pandemic relies on a trustworthy government reacting early, quickly and comprehensively to reliable and knowledgeable expert advice, to the threat with the right measures (including adequate financial support for those unable to work during lockdown restrictions), together with a population that tolerates and complies with these measures conscientiously and thoroughly.

### 3.5. Level of government trust

One of the recurring themes in those countries where the control of the virus was intermittent or generally poor overall was related to the degree to which populations trusted their governments. This was not just on the information describing on what the virus was, how it was spread, how it caused disease, and how to protect oneself from infection, but also about how governments communicated these ideas and risks, how they would combat fake news and misinformation, how they would protect those vulnerable to abuse during government-mandated lockdowns, and how they would compensate people if national pandemic public health restrictions meant that they could no longer go out to work, attend school or university, etc.

For example, those who could not work from home were concerned about the level of government financial support they would receive to replace their lost salaries. These job retention or ‘furlough;’ schemes were complex and varied between countries, but ranged from 40% to 80% salary replacement up to a certain absolute amount, sometimes with the balance being paid by the employer [178,285–288]. Those who were not eligible for these job retention payments due to the nature of their work, particularly in low income countries, were more likely to break stay-at home, self-isolation and self-quarantine rules, if they had to still go out to earn enough to be able to feed, clothe and house their families through any government-imposed pandemic confinement period [237,289].

As described above, in some South American countries, people often relied on conventional (television, radio and newspaper) and social (internet) media rather than their governments for timely and reliable advice on how to protect themselves from the virus, e.g. with masking and social distancing [248]. In some Western countries, initially, there was a lot of government distrust and/or compliance with social distancing, masking and any potential national lockdown measures—particularly where high level politicians and advisors were seen to openly violate such guidance themselves [290–294].

**Figure 3.**
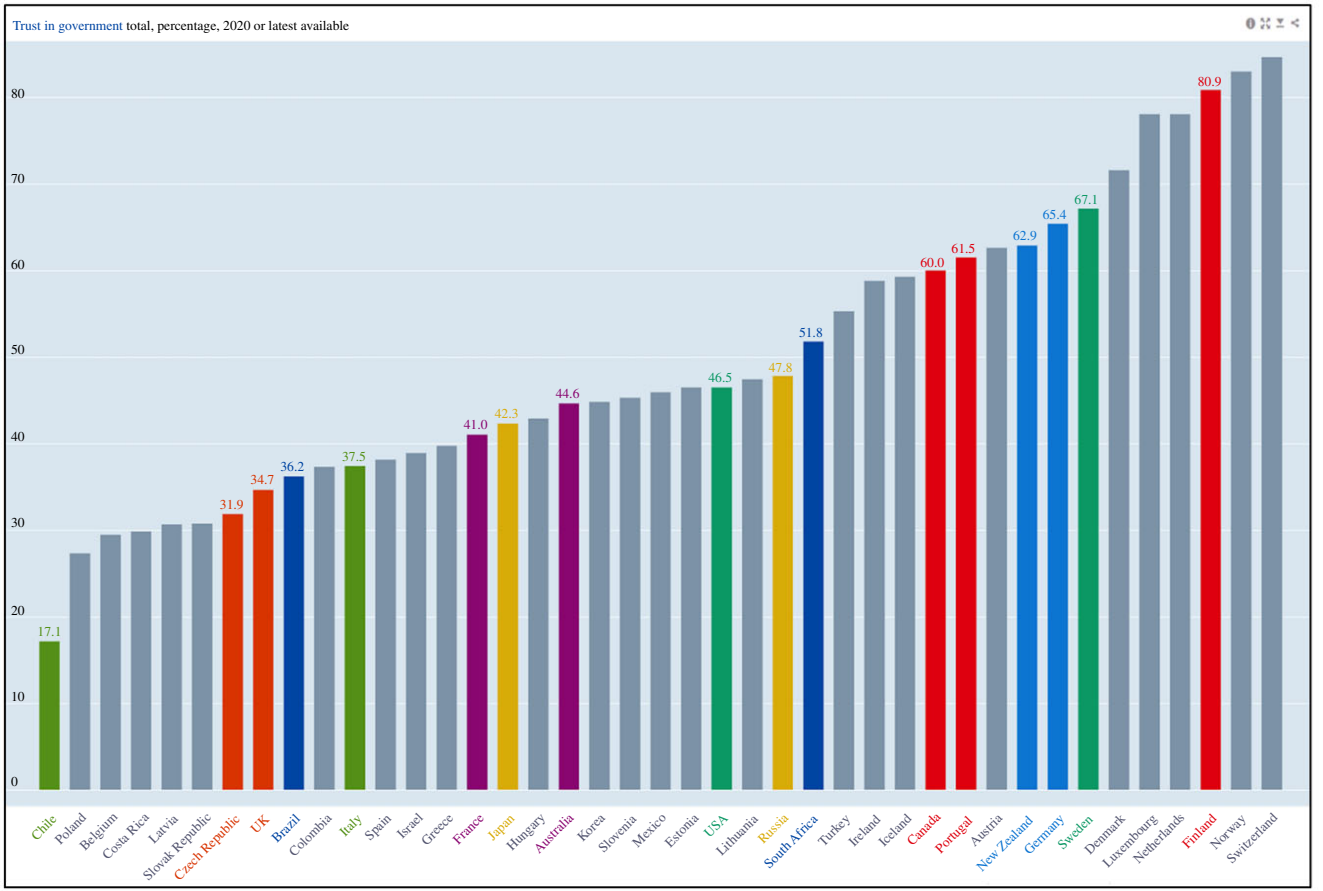
Trust in Government comparison (generated from [295]). Definition of ‘trust in government’ was ascertained by a simple survey question: ‘In this country, do you have confidence in … national government?’ Responses indicated on the vertical axis are a percentage of all survey respondents, pooled for the period 2010–2018 to improve the accuracy of the estimates, and designed to be nationally representative of the population aged 15 years and older.

One website compares the levels of government trust across multiple countries, where the definition of ‘trust in government’ was ascertained by a simple survey question ‘In this country, do you have confidence in… national government?’ (figure 3). Responses were pooled for the period 2010–2018 (so prior to the COVID-19 pandemic) to improve the accuracy of the estimates, and designed to be nationally representative of the population aged 15 years and older [295]. Asia, MENA and African countries are under-represented in this graphic. Note that of the 41 countries shown, with the exception of New Zealand, all the countries in the top quarter are Western European. The only non-European countries in the top half are USA, Canada, South Africa and New Zealand. Although most countries in the bottom half are from South America and Central/Eastern Europe, this also includes UK, Spain, France and Italy. Other non-European countries in the bottom half are Australia, Japan, South Korea and Israel.

Government trust by their populations is clearly a very difficult factor to change, particularly over the very short time-span of an early pandemic. Also, government trust is not the whole story as countries that initially controlled the pandemic well or poorly displayed a widely varying degree of trust in their respective governments.

## 4. Conclusion and recommendations

From the discussions above, it now seems that any attempt to design a single, early, effective global consensus on how to manage the next pandemic will be extremely difficult, perhaps impossible, e.g. we cannot say to a country that can barely feed its population nor provide clean drinking water that they need to be able to reach a daily testing capacity of 1000 test per million population withina few weeks or months. Thus, a more tiered approach, tailored to individual countries with similar characteristics (resources, competing priorities, government style and previous experience and expertise of dealing with emerging infections), will probably be more suitable and workable.

There are both government as well as population-related factors that need to be considered and even within a tiered approach to pandemic preparedness, different countries will have different ‘distances’ to travel to reach such tiered preparedness levels. If this tiered preparedness approach can be brought together and adopted under a trusted body, for example the WHO (though their decision-making processes are also relatively slow), which could offer any additional legislative, social and financial support, a more coordinated, tiered global response may be achieved—though this may also take time.

In this context, it seems reasonable to persuade the Western countries who may be in the ‘top’ tier of any such pandemic preparedness system, to follow the lead shown by many LMIC countries that reacted quickly and comprehensively in the early pandemic with NPIs because they were already aware that their fragile healthcare systems could not cope with surges in new COVID-19 cases. In this scenario, Western countries would have to suppress their concerns over the economy, education and civil liberties, *temporarily*, to get ahead of the virus to prevent it seeding large numbers of its populations—perhaps by a limited or more comprehensive form of local or national lockdown—depending how early they are able to detect its entry and spread. This type of drastic action would need substantial government economic support, which only these richer Western countries would be able to afford.

Finally, any tiered pandemic response led by national governments will require a high degree of acceptance and cooperation from their citizens. To achieve this and gain the trust of their people, national governments need to develop rapid cross-party mechanisms for more inclusive decision-making, engaging with representatives from health and social care, industry and commerce, childcare and education, hospitality and travel sectors—to gain this trust from their citizens for a more unified national and global response. To be effective, however, such negotiations would need to be completed within a few days, rather than weeks.

At the time of writing, the COVID-19 pandemic continues to evolve, with the recent emergence of the omicron variant. We acknowledge that national pandemic responses have also evolved alongside this, and the issues explored in this article are confined to the very early pandemic responses during February–July 2020.

## Data accessibility

Data extracted and used to plot figures 1 and 2 are provided as electronic supplementary material.

The data are provided in electronic supplementary material [296].
